# In vivo mapping of pharmacologically induced functional reorganization onto the human brain’s neurotransmitter landscape

**DOI:** 10.1126/sciadv.adf8332

**Published:** 2023-06-14

**Authors:** Andrea I. Luppi, Justine Y. Hansen, Ram Adapa, Robin L. Carhart-Harris, Leor Roseman, Christopher Timmermann, Daniel Golkowski, Andreas Ranft, Rüdiger Ilg, Denis Jordan, Vincent Bonhomme, Audrey Vanhaudenhuyse, Athena Demertzi, Oceane Jaquet, Mohamed Ali Bahri, Naji L. N. Alnagger, Paolo Cardone, Alexander R. D. Peattie, Anne E. Manktelow, Draulio B. de Araujo, Stefano L. Sensi, Adrian M. Owen, Lorina Naci, David K. Menon, Bratislav Misic, Emmanuel A. Stamatakis

**Affiliations:** ^1^ Division of Anaesthesia, University of Cambridge, Cambridge, UK.; ^2^ Department of Clinical Neurosciences, University of Cambridge, Cambridge, UK.; ^3^ Leverhulme Centre for the Future of Intelligence, University of Cambridge, Cambridge, UK.; ^4^ The Alan Turing Institute, London, UK.; ^5^ McConnell Brain Imaging Center, Montreal Neurological Institute, McGill University, Montreal, QC, Canada.; ^6^ Psychedelics Division - Neuroscape, Department of Neurology, University of California San Francisco, San Francisco, CA, USA.; ^7^ Center for Psychedelic Research, Department of Brain Sciences, Imperial College London, London, UK.; ^8^ Department of Neurology, Klinikum rechts der Isar, Technical University Munich, München, Germany.; ^9^ School of Medicine, Department of Anesthesiology and Intensive Care, Technical University of Munich, Munich, Germany.; ^10^ Department of Neurology, Asklepios Clinic, Bad Tölz, Germany.; ^11^ Department of Anaesthesiology and Intensive Care Medicine, Klinikum rechts der Isar, Technical University Munich, München, Germany.; ^12^ University of Applied Sciences and Arts Northwestern Switzerland, Muttenz, Switzerland.; ^13^ Department of Anesthesia and Intensive Care Medicine, Liege University Hospital, Liege, Belgium.; ^14^ Anesthesia and Perioperative Neuroscience Laboratory, GIGA-Consciousness Thematic Unit, GIGA-Research, Liege University, Liege, Belgium.; ^15^ GIGA-Cyclotron Research Centre-In Vivo Imaging, University of Liege, Liege, Belgium.; ^16^ Brain Institute, Federal University of Rio Grande do Norte, Natal, RN, Brazil.; ^17^ Department of Neuroscience and Imaging and Clinical Science, Center for Advanced Studies and Technology, Institute for Advanced Biomedical Technologies, University "G. d'Annunzio" Chieti-Pescara, Chieti, Italy.; ^18^ Institute for Memory Impairments and Neurological Disorders, University of California-Irvine, Irvine, CA, USA.; ^19^ Department of Psychology and Department of Physiology and Pharmacology, Western Institute for Neuroscience (WIN), Western University, London, ON, Canada.; ^20^ Trinity College Institute of Neuroscience, School of Psychology, Trinity College Dublin, Dublin, Ireland.; ^21^ Wolfon Brain Imaging Centre, University of Cambridge, Cambridge, UK.

## Abstract

To understand how pharmacological interventions can exert their powerful effects on brain function, we need to understand how they engage the brain’s rich neurotransmitter landscape. Here, we bridge microscale molecular chemoarchitecture and pharmacologically induced macroscale functional reorganization, by relating the regional distribution of 19 neurotransmitter receptors and transporters obtained from positron emission tomography, and the regional changes in functional magnetic resonance imaging connectivity induced by 10 different mind-altering drugs: propofol, sevoflurane, ketamine, lysergic acid diethylamide (LSD), psilocybin, N,N-Dimethyltryptamine (DMT), ayahuasca, 3,4-methylenedioxymethamphetamine (MDMA), modafinil, and methylphenidate. Our results reveal a many-to-many mapping between psychoactive drugs’ effects on brain function and multiple neurotransmitter systems. The effects of both anesthetics and psychedelics on brain function are organized along hierarchical gradients of brain structure and function. Last, we show that regional co-susceptibility to pharmacological interventions recapitulates co-susceptibility to disorder-induced structural alterations. Collectively, these results highlight rich statistical patterns relating molecular chemoarchitecture and drug-induced reorganization of the brain’s functional architecture.

## Teaser

Anaesthetics and psychedelics systematically engage diverse neurotransmitter systems to perturb consciousness.

## INTRODUCTION

Understanding how the brain orchestrates complex signals across spatial and temporal scales to support cognition and consciousness is a fundamental challenge of contemporary neuroscience. By inducing profound but reversible alterations of brain function, psychoactive compounds provide neuroscientists with the means to manipulate the brain without requiring surgical intervention. In combination with noninvasive brain imaging techniques such as functional magnetic resonance imaging (fMRI), acute pharmacological interventions have therefore emerged as a prominent tool for causal investigation of the relationship between brain and cognitive function in healthy humans (*1*).

Mind-altering pharmacological agents also play a fundamental role in modern clinical practice. The invention of anesthesia was a major milestone in medical history, enabling millions of life-saving surgeries to take place every year (*2*). Other drugs that influence the mind without suppressing consciousness, such as the cognitive enhancers modafinil and methylphenidate, have found useful applications in alleviating the cognitive symptoms of syndromes such as attention deficit/hyperactivity disorder, narcolepsy, and traumatic brain injury (TBI) (*3*–*6*). More recently, classic and “atypical” psychedelics are increasingly being investigated for their potential to provide breakthrough avenues to treat psychiatric conditions, with recent successes in clinical trials holding promise to help mitigate the current scarcity of therapies for treatment-resistant depression and other neuropsychiatric disorders (*7*–*12*). For these convergent reasons, the effects of anesthetics, psychedelics, and cognitive enhancers on brain function are becoming the focus of the intense investigation, revealing both similarities and differences between them (*13*–*20*).

Pharmacological agents exert their mind-altering effects by tuning the brain’s neurotransmitter landscape. Neurotransmitters engage receptors on neurons’ membranes to mediate the transfer and propagation of signals between cells, modulate the functional configurations of neuronal circuits, and ultimately shape network-wide communication (*21*–*23*). Several psychoactive drugs appear to exert their effects on the mind and brain primarily through one or few specific neurotransmitters (*2*): The main action of the general anesthetics propofol and sevoflurane is the agonism of γ-aminobutyric acid type A (GABA_A_) receptors, with additional attenuation of glutamatergic synaptic signaling [mediated by both AMPA and *N*-methyl-d-aspartate (NMDA) receptors] (*24*–*28*). Ketamine (a dissociative anesthetic at high doses and atypical psychedelic at low doses) is an NMDA receptor antagonist (*29*–*31*); the classic psychedelics lysergic acid diethylamide (LSD), psilocybin, and N,N-Dimethyltryptamine (DMT) are agonists of the serotonin 2A receptor, with a strong dependence between subjective efficacy and 2A receptor affinity (*32*–*34*).

However, in the words of Sleigh and colleagues, “Linking observed molecular actions for any particular drug with its clinical effects is an abiding pharmacological problem” (*35*): knowing the primary molecular target is not sufficient to understand a drug’s effects on the brain function, for several reasons. First, given the brain’s intricate, nested feedback loops and recurrent pathways of connectivity, even a relatively selective drug can end up influencing unrelated systems beyond what may be apparent from in vitro studies. Second, most mind-altering compounds are also known to have an affinity for other receptors. Evidence has been accumulating that multiple neurotransmitter influences may be involved in both the neural and subjective experiences induced by many consciousness-altering drugs. In the last years, human neuroimaging studies identified the involvement of the dopaminergic system in both propofol-induced anesthesia (*36*) and the subjective effects of LSD (*37*). More broadly, a recent large-scale study, combining receptor expression from transcriptomic data with linguistic processing of several thousand subjective reports of psychedelic use, identified complex multivariate patterns of association between neurotransmitters and their effects on the mind elicited by a wide variety of psychedelics, even for putatively selective agents (*38*). At the same time, molecularly different compounds can exert similar effects on both the mind and brain: for instance, LSD and (subanesthetic) ketamine can produce subjectively similar effects and changes in terms of structure-function coupling and the complexity of brain activity—despite acting on different pathways (*39*). This suggests both divergent and convergent effects of different pharmacological agents on the brain’s rich neurotransmitter landscape.

Last, the human brain exhibits rich patterns of anatomical, functional, cytoarchitectonic, and molecular variations (*40*–*44*). These patterns also extend to the regional distribution of different neurotransmitter receptors and transporters, which vary widely not only in terms of their affinity, time scales, and downstream effects on neuronal excitability but also in terms of their distribution across regions, layers, and neuron types (*21*, *22*, *45*). Therefore, our knowledge of how a drug influences neurotransmission must take into account the neuroanatomical distribution of its target neurotransmitters—an essential step toward explaining how different neurotransmitters mediate the capacity of different drugs to shape the functional and computational properties of the brain’s architecture (*21*, *23*, *46*).

Here, we sought to address this question in a data-driven way, mapping the neurotransmitter landscape of drug-induced alterations in the brain’s functional connectivity (FC). To do so, we leveraged two unique datasets: (i) a recently assembled collection of in vivo maps of regional expression from 19 different receptors and transporters, obtained from positron emission tomography (PET) scanning of more than 1200 total individuals, providing the most detailed information about neuromodulators and their spatial distribution available to date (*23*); and (ii) resting-state fMRI (rs-fMRI) data acquired under the effects of the serotonergic psychedelics LSD (*47*), psilocybin (*48*), DMT (*49*), ayahuasca (*50*), and 3,4-methylenedioxymethamphetamine (MDMA) (*51*); subanesthetic doses of ketamine (acting as an “atypical psychedelic”) (*52*) as well as anesthetic doses (acting as a “dissociative anesthetic”); the cognitive enhancers modafinil (*53*) and methylphenidate (*6*); and the anesthetics sevoflurane (*54*) and propofol (*55*, *56*) (which we compared against preanesthetic baseline and postanesthetic recovery); representing a total of 382 sessions of pharmacological MRI from 224 distinct subjects and 10 distinct pharmacological agents. Through pharmacologically modulated rs-fMRI, we can characterize a drug’s effects on the brain’s spontaneous activity, without the interference of any specific task (*1*).

Thus, our goal was to obtain a comprehensive mapping between the cortical distributions of neurotransmitters and a set of diverse psychoactive pharmacological agents (covering the range from anesthetics to psychedelics), in terms of their effects of FC. There have been other studies looking at the relationships between brain changes induced by one or few psychoactive drugs, and one or few neurotransmitter systems (*36*, *37*, *57*–*62*), and a previous effort considering how changes in cerebral blood flow induced by different psychiatric medications depend on the distribution of receptors (*63*). However, to our knowledge, this is the largest fMRI study to date not only in terms of the number, variety, and potency of psychoactive pharmacological agents included but also in terms of the breadth and coverage of neurotransmitter systems considered.

## RESULTS

### Characterization of drug-induced functional reorganization and receptor distributions

**Fig. 1.**
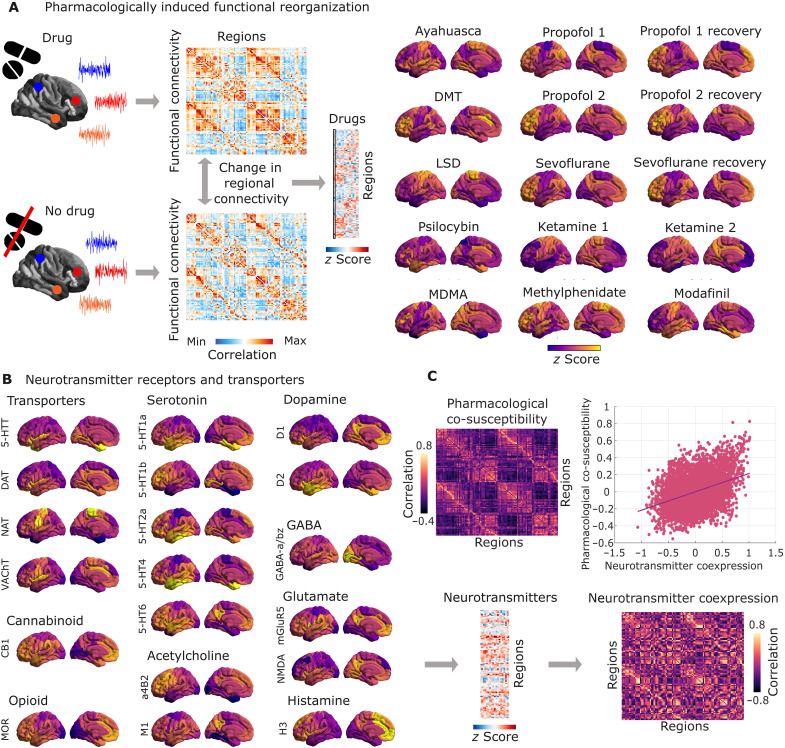
Overview of receptors and pharmacological rs-fMRI data. (**A**) For each psychoactive drug, its pattern of pharmacologically induced functional reorganization is quantified as the average (across subjects) of the within-subject difference in regional FC weighted degree (sum of each region’s positive connections) between task-free fMRI scans at baseline and under the drug’s effects. The result is a map of 100 cortical regions with 15 drug-related contrasts. (**B**) Neurotransmitter systems are mapped with PET with radioligands for 15 receptors and 4 transporters, resulting in a map of 100 cortical regions with 19 neurotransmitters. (**C**) A region-by-region matrix of pharmacological co-susceptibility is obtained by pairwise correlation of the regional patterns of drug-induced FC changes across all concatenated subject-wise delta maps. A region-by-region matrix of neurotransmitter coexpression is obtained by pairwise correlation of the regional patterns of neurotransmitter expression, concatenated across all 19 receptor and transporter PET maps. These two matrices are significantly correlated (Spearman’s correlation across *N* = 4950 edges) even after removing the exponential relationship with Euclidean distance between regions.

To establish a relationship between neurotransmitter systems and pharmacologically induced reorganization of the brain’s functional architecture, we combine two sets of neuroimaging data, each collected from multiple studies. On one hand, we characterize drug-induced functional reorganization as the changes in FC obtained by contrasting rs-fMRI at baseline and under the acute effect of a psychoactive drug. We considered the general anesthetics propofol (two independent datasets, both at a dosage sufficient to induce loss of responsiveness) and sevoflurane; the cognitive enhancers modafinil and methylphenidate; ketamine, acting as both atypical psychedelic (at subanesthetic doses) and as a dissociative anesthetic (*18*, *31*); and the serotonergic psychedelics LSD, psilocybin, DMT, ayahuasca, and MDMA (Fig. 1). For sevoflurane and both propofol datasets, we considered two contrasts: drug versus pre-induction baseline, and drug versus postanesthetic recovery (recovery data were not available for ketamine). We followed the same preprocessing and denoising procedure for each dataset, to ensure comparability (see Supplementary Methods and table S1).

On the other hand, we consider the cortical distribution of 15 neurotransmitter receptors and 4 transporters, obtained from in vivo PET (*23*). Overall, nine neurotransmitter and neuromodulatory systems (“neurotransmitters” for short) are covered: dopamine (D1, D2, and DAT), noradrenaline (NAT), serotonin (5-HT1A, 5-HT1B, 5-HT2A, 5-HT4, 5-HT6, and 5-HTT), acetylcholine (α4β2, M1, and VAChT), glutamate (mGluR5 and NMDA), GABA (GABA_A_), histamine (H3), cannabinoid (CB1), and opioid (MOR) (Fig. 1, A and B) (*23*). Both rs-fMRI and PET maps were parcellated into 100 functionally defined regions according to the Schaefer atlas (*64*).

### Shared chemoarchitecture and shared response to pharmacological perturbations

Receptors and transporters shape the way that neurons respond to neurotransmission and neuromodulatory influences. In turn, psychoactive drugs exert their effects (primarily) by acting on neurotransmitters and neuromodulators. Therefore, we reasoned that everything else being equal, regions that express similar patterns of receptors and transporters should exhibit similar patterns of susceptibility to drug-induced functional reorganization.

To address this question, we computed matrices of pharmacological co-susceptibility and neurotransmitter coexpression between pairs of regions, by correlating respectively the regional patterns of drug-induced FC changes (across all concatenated subject-wise delta maps) and the regional patterns of neurotransmitter expression (across all 19 receptor and transporter PET maps). To account for spatial autocorrelation in molecular and FC attributes, we regressed out from both matrices the exponential trend with Euclidean distance (*65*).

Supporting our hypothesis, we found that pharmacological co-susceptibility is significantly correlated with neurotransmitter profile similarity: the extent to which two regions’ FC patterns are similarly affected by perturbations induced by different psychoactive drugs, is predicted by the extent to which they coexpress neurotransmitter receptors and transporters: ρ = 0.34, *P* < 0.001 after regressing out the effects of Euclidean distance (Fig. 1C). In other words, regions that exhibit shared chemoarchitecture also respond similarly across pharmacological perturbations. To account for potential confounds due to partial volume effects, we repeated this analysis but instead of regressing out exponential distance, we regressed out a matrix of similarity between regions’ nongray-matter tissue probability (see Supplementary Methods and fig. S1).

### Multivariate receptor-drug associations

The previous analysis revealed a relationship between large-scale patterns of neurotransmitter expression, and large-scale patterns of functional susceptibility to pharmacological perturbations, complementing previous work that identified relationships between individual drugs and individual receptors. However, neither of these two approaches captures the full richness of the two datasets used here. To obtain a synthesis between these two approaches, we used a multivariate association technique, partial least squares correlation [PLS; also known as a projection to latent structures (*66*)], which enabled us to identify multivariate patterns of maximum covariance between drug-induced effects on FC, and the cortical distributions of neurotransmitter expression (*67*).

**Fig. 2.**
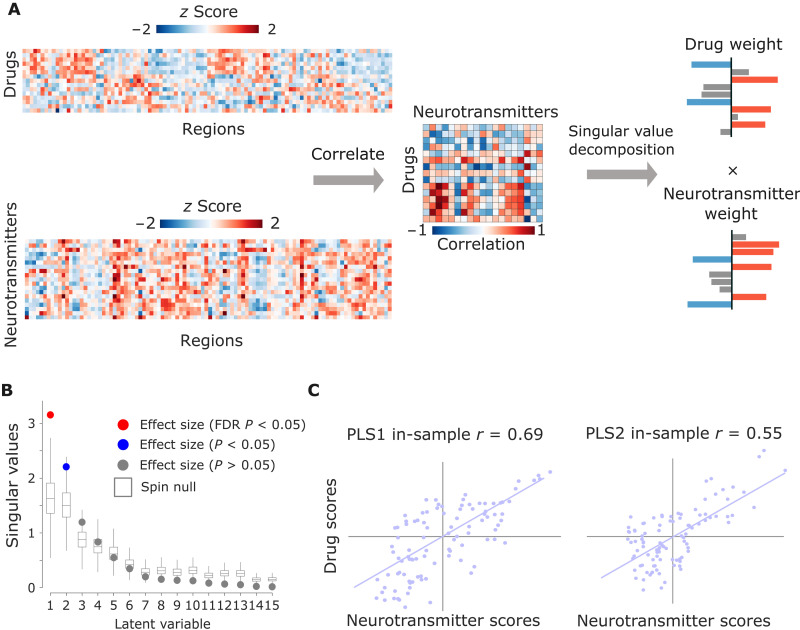
PLS analysis reveals spatially covarying patterns of pharmacologically induced functional reorganization and neurotransmitter expression. (**A**) PLS analysis relates two data domains by correlating the variables across brain regions and subjecting this to singular value decomposition. This results in multiple latent variables: linear weighted combinations of the original variables (neurotransmitter weights and drug weights) that maximally covary with each other. (**B**) Latent variables are ordered according to effect size (the proportion of covariance explained between neurotransmitter expression and drug-induced functional reorganization they account for) and shown as colored dots. (**C**) The first two latent variables (PLS1 and PLS2) were statistically significant, with respect to the spatial autocorrelation-preserving null model shown in gray (10,000 permutations), with PLS1 also surviving FDR correction for multiple comparisons. The first latent variable accounted for 57% of covariance, and the second latent variable accounted for 28%. Neurotransmitter (drug) scores are defined as the projection of the original neurotransmitter density (drug-induced FC changes) matrix onto the neurotransmitter (drug) weights, such that each brain region is associated with a neurotransmitter and drug score. By design, neurotransmitter and drug scores correlate highly. Both PLS1 and PLS2 were also identified as statistically significant when tested against an alternative null model, whereby participants’ conditions (drug versus no drug) were randomly permuted before averaging, repeating this procedure 1000 times (fig. S3).

This analysis indicated the presence of two statistically significant latent variables (linear weighted combinations of the original variables) relating pharmacologically induced functional reorganization to neurotransmitter profiles, together accounting for nearly 85% of covariance. Significance was assessed against autocorrelation-preserving spin-based null models, embodying the null hypothesis that drug effects and neurotransmitters are spatially correlated with each other purely because of inherent spatial autocorrelation (*68*, *69*), followed by false discovery rate (FDR) correction for multiple comparisons (Fig. 2). The first latent variable remained significant even after FDR correction. We further cross-validated this result against spatial dependence by fitting a model on a training set of spatially adjacent brain regions, and testing the model on a held-out set of regions that are as far from the training set nodes as possible, and therefore likely divergent in annotation properties; out-of-sample *r* = 0.46 for PLS1 and 0.54 for PLS2, both *P* < 0.001 from *t* test against spin-based null distributions (fig. S2).

**Fig. 3.**
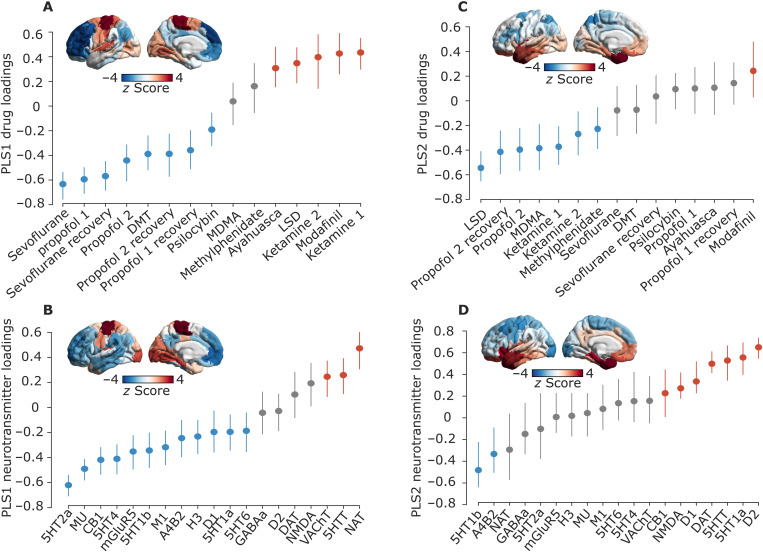
PLS scores and loadings from significant latent variables. (**A** and **B**) Scores and loadings for PLS1. (**C** and **D**) Scores and loadings for PLS2. Brain plots: Drug scores (top row) and neurotransmitter scores (bottom row) for each brain region are obtained by projecting the original neurotransmitter and drug data back onto the PLS analysis–defined drug/neurotransmitter weights, indexing the extent to which a brain region expresses covarying drug/neurotransmitter patterns. In turn, neurotransmitter (drug) loadings are defined as Pearson’s correlation between each neurotransmitter’s density of expression (drug-induced FC changes) across brain regions and the PLS analysis–derived score pattern and plotted as *z* scores. Error bars indicate a 95% confidence interval (CI), and color indicates the direction of the effect: positive (orange), negative (blue), or null (gray). Same-colored loadings and scores covary positively, whereas opposite-colored drugs and scores covary negatively. The label “ketamine 1” refers to the subanesthetic dose, and “ketamine 2” is the higher (anesthetic) dose. The label “propofol 1” refers to the Cambridge dataset, and “propofol 2” is the Western dataset.

For each latent variable, each brain region is associated with a neurotransmitter and drug score. In turn, neurotransmitter (drug) loadings are defined as the correlation between the PLS-derived score pattern and each neurotransmitter’s density of expression (resp., drug-induced FC changes) across brain regions. Taking into account the first latent variable (PLS1), drug loadings showed a distinction of pharmacological effects into two groups, with all anesthetics (except ketamine) on one side, and both ketamine datasets dominating the opposite side, together with LSD, ayahuasca, and modafinil (Fig. 3A). Neurotransmitter loadings divided the receptors from transporters: at the positive end (orange), the noradrenaline, serotonin, and acetylcholine transporters [with the dopamine transporter following closely, but narrowly including zero in its 95% confidence interval (CI)]; all receptors except NMDA were instead at the negative end (blue), although some included zero in their CI (Fig. 3B).

Pertaining to the second latent variable (PLS2), neurotransmitter loadings mainly identified a monoamine-rich end (with dopamine and serotonin), although 5-HT1b occupied the opposite end. However, the drug loadings were less clearly discernible, with modafinil alone at one end, and a mixture of propofol, psychedelics, and both ketamine datasets at the other end. Both neurotransmitter and drug scores markedly separated dorsal and ventral aspects of the brain for this second latent variable (Fig. 3).

### Alignment of pharmacologically induced alterations with functional, anatomical and molecular hierarchies

Neurotransmitter and drug scores (whose spatial similarity PLS is designed to maximize) provide information about the regional distribution of neurotransmitter-drug associations. Neurotransmitters and drugs whose regional distribution correlates positively with the score pattern covary with one another in the positively scored regions, and vice versa for negatively scored regions.

**Fig. 4.**
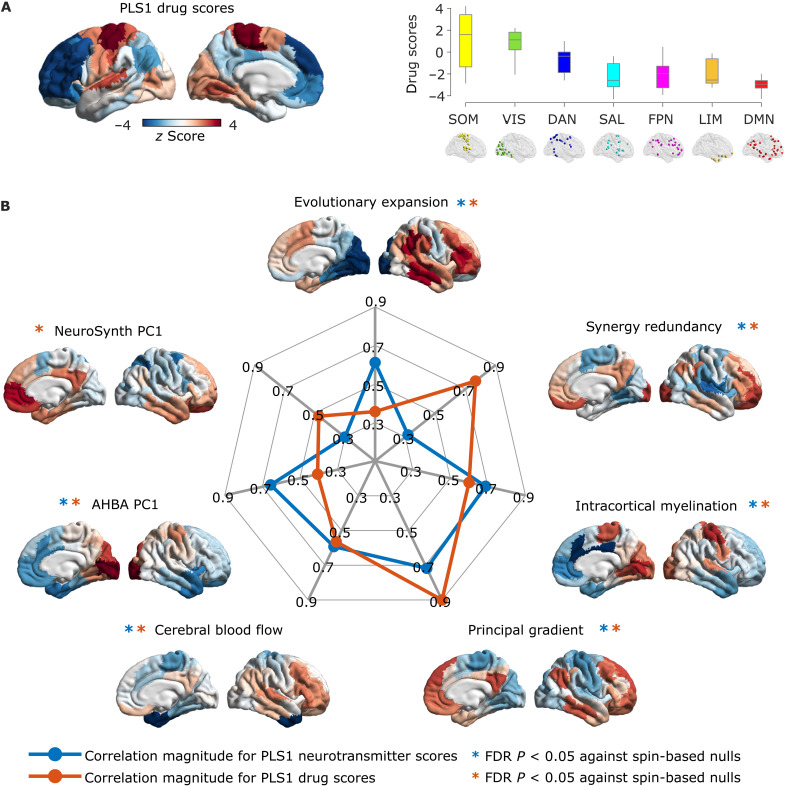
Correspondence between the principal axis of drug-neurotransmitter scores and functional, anatomical, and molecular hierarchies. (**A**) Cortical distribution of drug scores for PLS1, and their association with intrinsic resting-state networks. (**B**) The radial plot represents the absolute value of Spearman’s spatial correlation between PLS1 drug and neurotransmitter scores, and each of the seven cortical hierarchies obtained from different neuroimaging modalities (note that the myelin and AHBA PC1 maps are reversed with respect to the remaining hierarchies). *, FDR-corrected *P*_spin_ < 0.05. Results were replicated after regressing out of PLS1 drug and neurotransmitter scores the regional prevalence of nongray matter tissue (fig. S6).

PLS1 scores correspond to the main axis of covariance between neurotransmitter expression and pharmacologically induced functional reorganization. For both drug and receptor scores, we observed that their regional distribution reflected the brain’s organization into intrinsic resting-state networks (RSNs) (*70*), setting apart visual and somatomotor cortices from association cortices (Figs. 3 and 4). It is possible that the correspondence of PLS1 scores with RSNs may be in part driven by the fact that these networks are predicated in terms of functional neuroimaging, which we also used to characterize drug-induced functional reorganization in our data. Therefore, we next sought to determine whether our data-driven topographic patterns reflect other cortical gradients of variation in terms of functional, anatomical, and molecular attributes. To this end, we considered intracortical myelination obtained from T1w/T2w MRI ratio (*43*), evolutionary cortical expansion obtained by comparing human and macaque (*71*), the principal component of variation in gene expression from the Allen Human Brain Atlas transcriptomic database (“AHBA PC1”) (*44*, *72*), the principal component of variation in task activation from the NeuroSynth database (“NeuroSynth PC1”) (*44*, *73*), and the principal gradient of FC (*42*). Since pharmacological interventions exert their effects on the brain via the bloodstream, we also included a map of cerebral blood flow (*40*). Last, we included a recently derived gradient of the regional prevalence of different kinds of information, from redundancy to synergy (*74*).

We observed significant correlations (assessed against spin-based null models and corrected for multiple comparisons) between each cortical hierarchy and both neurotransmitter and drug scores for PLS1 (except for PLS1 neurotransmitter scores versus NeuroSynth PC1; FDR-corrected *P*_spin_ = 0.056) (Fig. 4). The scores for PLS2 instead identified a ventral-dorsal pattern of regional variation (Fig. 3 and fig. S4), which did not significantly correlate with any of the canonical gradients of a hierarchical organization (all FDR-corrected *P >* 0.05 against spin-based null models). As an alternative avenue to characterize this second, nonhierarchical cortical pattern, we quantified its spatial similarity with 123 brain maps pertaining to cognitive and psychological processes, obtained from NeuroSynth. Significant FDR-corrected correlations (assessed against spin-based null models) were observed for both PLS2 drug and neurotransmitter scores, in particular pertaining to emotion-related terms (“emotion” and “valence”) on the positive end—consistent with the involvement of limbic and salience networks in PLS2 (fig. S4).

By comparing the score pattern to multiple cortical hierarchies, we demonstrate that the relationship between psychoactive drug effects and neurotransmitter receptor densities fits into the broader sensory-association hierarchy of the cortex (*40*). Given the presence of multiple significant predictors for PLS1 drug and neurotransmitter scores, we then applied dominance analysis (see Materials and Methods) to take into account all cortical hierarchies simultaneously (fig. S5). For PLS1 neurotransmitter scores, we found relatively even contributions from the various hierarchies, whereas, for the PLS1 drug scores, the principal gradient of FC was the predictor with the greatest percentage of relative importance (fig. S5).

### Neurotransmitter landscape of pharmacologically induced functional reorganization

**Fig. 5.**
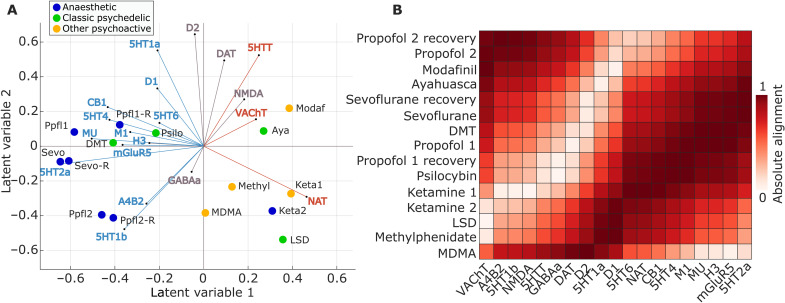
Joint mapping of neurotransmitters and pharmacological agents. (**A**) Biplot: Each drug is represented as a point reflecting its projection onto the first two latent variables of the PLS analysis, color-coded based on its effects on subjective experience (anesthetic, psychedelic, or other psychoactive). Each neurotransmitter receptor and transporter is represented as a vector in the same two-dimensional space defined by the first two latent variables, color-coded by loading onto PLS1 as shown in Fig. 3 (orange for positive; blue for negative; and gray if the 95% CI intersects zero). For both propofol (Ppfl) and ketamine (keta), the number refers to the dataset, with 1 identifying the weaker dose, and 2 identifying the stronger dose. A qualitatively similar mapping between drugs and neurotransmitters on the first two latent variables is observed when the methylphenidate dataset (which was the only dataset obtained from patients rather than healthy controls) is excluded (fig. S7). Figure S8 shows the drugs and neurotransmitters separately. (**B**) Alternative representation of the mapping between neurotransmitters and pharmacological agents in the same space: Each cell in the heatmap indicates the alignment between the corresponding drug and neurotransmitter (absolute cosine of the angle between their vectors, in the space of the first two latent variables).

Taking into account the first two PLS latent variables shows how each drug-specific pattern of pharmacologically induced functional reorganization can be interpreted in terms of contributions from different receptors (note that sign is arbitrary) (Fig. 5A; quantification of the respective alignment between each drug and each neurotransmitter in the joint space is provided in Fig. 5B). As already shown in Fig. 3, the first latent variable revealed a stark division between transporters and receptors, which discriminates between traditional anesthetics and other psychoactive substances. In terms of pharmacological alterations, the non-monoaminergic end of the second latent variable loaded onto drugs with relatively stronger effects on subjective experiences (the higher doses of anesthetic, including ketamine, LSD, and MDMA). However, methylphenidate and subanesthetic ketamine also loaded onto this end of the second latent variable. Altogether, we find that the first latent variable captures a strong relationship between drug interventions and receptor systems, that is both biologically relevant and aligns with the functional organization of the brain.

### Co-susceptibility to pharmacological and pathological alterations

Last, we wondered if the functional co-susceptibility of different regions to transient pharmacological perturbations may provide a functional proxy for their co-susceptibility to structural perturbations resulting from different neurological, neurodevelopmental, and psychiatric disorders. To this end, we combined 11 spatial maps of cortical thickness abnormalities made available by the Enhancing Neuroimaging Genetics through Meta-Analysis (ENIGMA) consortium (*65*, *75*, *76*): 22q11.2 deletion syndrome, attention-deficit/hyperactivity disorder, autism spectrum disorder, idiopathic generalized epilepsy, right temporal lobe epilepsy, left temporal lobe epilepsy, depression, obsessive-compulsive disorder, schizophrenia, bipolar disorder, and Parkinson’s disease. For simplicity, we refer to diseases, disorders, and conditions as “disorders” throughout the text. The cortical abnormality maps summarize contrasts between more than 17,000 adult patients and 22,000 controls collected following identical processing protocols to ensure maximal comparability (*75*, *76*).

**Fig. 6.**
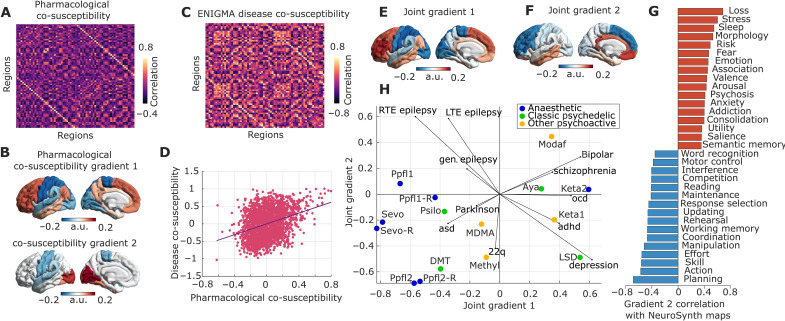
Co-susceptibility to pharmacological and pathological alterations. (**A**) Similarity of regional susceptibility to pharmacological alterations (same as Fig. 1C, but for the Desikan-Killiany atlas). (**B**) First two principal gradients of regional susceptibility to pharmacological alterations obtained from diffusion map embedding. (**C**) Regional co-susceptibility to neuropsychiatric and neurological alterations of cortical thickness. (**D**) Brain regions that are similarly affected by pharmacology, in terms of functional reorganization, are also similarly affected across disorders, in terms of cortical thickness abnormalities (Spearman’s correlation across *N* = 2278 edges). This relationship persists after regressing out the exponential trend with Euclidean distance, and also after regressing out the matrix of similarity of regional nongray matter tissue probability (fig. S11). (**E** and **F**) First two principal gradients of regional joint susceptibility to pharmacological and neuropsychiatric and neurological alterations obtained from diffusion map embedding. Together, these first two gradients account for nearly half of the variation in regional co-susceptibility (fig. S10). (**G**) Significant spatial correlations (Spearman’s ρ) between gradient 2 and 123 term-based meta-analytic maps from NeuroSynth. Significance is assessed against spatial autocorrelation-preserving null models and corrected for multiple comparisons using the FDR. (**H**) The plot shows each drug-induced functional reorganization map (colored dots) and each disease-associated cortical alteration map (vectors), in the space of the two principal gradients of joint susceptibility, obtained by correlating the corresponding cortical patterns. Adhd, attention deficit/hyperactivity disorder; asd, autistic spectrum disorder; ocd, obsessive-compulsive disorder; gen., generalized; RTE, right temporal lobe epilepsy; LTE, left temporal lobe epilepsy; a.u., arbitrary units.

Following the same procedure used to obtain the region-by-region matrices of pharmacological co-susceptibility and neurotransmitter coexpression (Fig. 1C and Fig. 6A), we obtained a region-by-region matrix of co-susceptibility to disorder-induced cortical abnormality by correlating the regional patterns of cortical abnormality across all 11 disorders (Fig. 6C) (*65*). Correlating this matrix of regional co-susceptibility to disease-associated perturbations against the previously derived matrix of regional co-susceptibility to pharmacological perturbations, we found a statistically significant relationship (Spearman’s ρ = 0.31, *P* < 0.001 after regressing out the effect of Euclidean distance) (Fig. 6D). This result goes beyond a recent demonstration that molecular similarity and disorder similarity are correlated (*65*), by showing that a correlation also exists between different kinds of perturbations: anatomical and pharmacological.

Having ascertained that susceptibility to pharmacological and pathological alterations is positively correlated, we next sought to identify a low-dimensional representation of this joint co-susceptibility. Whereas the aim of PLS analysis is to enable a many-to-many mapping between variables (*67*), here, our aim was to find common patterns that describe how regions vary in their overall co-susceptibility to perturbations, rather than focusing on specific perturbations. In other words, this is a dimensionality reduction problem, rather than multivariate association. Therefore, we resorted to a nonlinear dimensionality reduction algorithm, diffusion map embedding (*42*, *77*), which enabled us to obtain joint gradients of variation from pharmacological and disease-associated co-susceptibility using a recently developed method for network fusion (*78*).

When applying diffusion map embedding to the matrix of pharmacological co-susceptibility only, we found that the first two gradients of variation in regional pharmacological susceptibility coincide with the two well-known principal gradients of FC identified by Margulies *et al.* (*42*) (Fig. 6B): the first gradient sets apart unimodal from transmodal cortices, whereas the second gradient is anchored in visual cortex at one end, and somatomotor cortex at the other end. This observation suggests that co-susceptibility to pharmacological perturbations recapitulates intrinsic functional architecture, as well as co-susceptibility to disorder-induced structural perturbations.

When considering the gradients obtained from regional joint susceptibility to pharmacological and neuropsychiatric alterations, we found that the main axis of variation coincides with the first gradient of pharmacological susceptibility, as well as the well-known principal gradient of FC (*42*), setting apart unimodal cortices (somatomotor and visual networks) from transmodal cortices (especially default and frontoparietal networks), reminiscent of the PLS1 scores (Fig. 6E and fig. S9A). The second gradient instead is anchored in anterior dorsolateral cortices at one end (especially pertaining to the frontoparietal network) and ventromedial and temporal cortices at the other end, spanning limbic and salience/ventral attention networks, reminiscent of the PLS2 scores (Fig. 6F and fig. S9B). To characterize this second cortical gradient of joint susceptibility, we quantified its spatial similarity with 123 NeuroSynth maps (Fig. 6G). We observed several significant FDR-corrected correlations (assessed against spin-based null models), including with valence and emotion maps, which had also emerged as significant correlates of the PLS2 drug and neurotransmitter scores—consistent with the prominent involvement of limbic and salience networks. In addition, however, the second gradient of joint pharmacological-pathological susceptibility correlated with meta-analytic maps pertaining to psychiatric conditions and symptoms, such as “loss,” “stress,” “fear,” “psychosis,” and “anxiety” (Fig. 6G), highlighting the relevance of this second gradient for pathology. Last, we show how disorders and pharmacological alterations map onto the space defined by the two joint gradients of co-susceptibility, by plotting the correlations between the gradients and each pattern of drug-induced functional reorganization and disorder-associated anatomical reorganization (Fig. 6H).

### Validation and additional analyses

To mitigate both physiological and motion-related confounds, our denoising pipeline included scrubbing and regression of 12 motion parameters and 10 principal components from white matter and cerebrospinal fluid. Nevertheless, since several of our pharmacological interventions were accompanied by significant differences in head motion (mean framewise displacement; table S2), we also repeated our analysis after implementing three different strategies for further controlling the potential confounding effects of motion. First, we regressed the FC change at each region observed in each dataset, against the standardized difference in mean framewise displacement observed for that dataset, as a way of further controlling for any differences in motion across datasets that may be confounding our analysis. The resulting PLS1 also primarily separated anesthetics from other psychoactive substances and neurotransmitter receptors versus transporters, as observed in our main analysis, with transporters covarying with cognitive enhancers and most psychedelics in primary sensory and motor regions, and receptors primarily covarying with traditional anesthetics in transmodal association cortices (fig. S12). PLS1 scores also remained significantly aligned with the cortical hierarchy after motion regression (fig. S13). Second, we replicated our results with low-motion subjects only. We show that analogous hierarchically organized mappings between different drug types and receptors versus transporters can be observed when any subjects with mean framewise displacement greater than 0.3 in either baseline or drug scan are excluded from the analysis (fig. S14). Third, we further show that our main results are replicated if we only include datasets for which the difference in mean framewise displacement between baseline and drug scans is not statistically significant (table S2), namely, the Cambridge propofol dataset, the Cambridge propofol recovery dataset, the psychedelic ketamine dataset, modafinil, methylphenidate, ayahuasca, MDMA, and psilocybin (which therefore include each type of drug: anesthetic, psychedelic, and cognitive enhancer). Again, we find that our results replicate what we observed in our main analysis, with a principal latent variable organized along the unimodal-transmodal axis separating receptors and transporters (fig. S15). The convergence of these three distinct approaches to motion correction in replicating the results of our main analysis demonstrates that our results are robust to the potential confounding effects of motion and remain stable even under conservative strategies for motion correction.

To further ensure the robustness of our main results, we also replicated them using different cortical parcellations: the anatomically defined Lausanne-114 (fig. S16) (*79*) and Schaefer-232, which combines 200 cortical and 32 subcortical regions (fig. S17). Both showed an analogous unimodal-transmodal PLS1 separating receptors from transporters and traditional anesthetics from other psychoactive drugs, and a ventromedial-dorsolateral pattern for PLS2. The positive association between neurotransmitter coexpression and pharmacological co-susceptibility was also preserved when subcortical structures were included (fig. S18). The subcortical portion of the PLS1 scores for the Schaefer-232 PLS analysis identified an anterior-posterior pattern, separating the caudate and putamen from the thalamus, hippocampus, and amygdala (fig. S17). A similar anterior-posterior division of subcortical structures (though with the anterior thalamus diverging from the posterior) was also observed when repeating the PLS analysis exclusively on the 32 subcortical regions of interest (ROIs) (fig. S19). This analysis revealed a monoamine- (dopamine- and serotonin-)dominated principal latent variable, positively associated with the drugs having the strongest effects (fig. S19).

## DISCUSSION

Here, we characterized how mind-altering pharmacological agents engage the brain’s rich neurotransmitter landscape to exert their effects on brain function. We developed a computational framework to relate the regional reorganization of fMRI FC induced by 10 different mind-altering drugs, and the cortical distribution of 19 neurotransmitter receptors and transporters obtained from PET (*23*). This approach allowed us to discover large-scale spatial gradients relating pharmacologically induced changes in FC to the underlying neurotransmitter systems. By relating microscale molecular chemoarchitecture and macroscale functional reorganization induced by drugs with potent acute effects on the mind, our results provide a first step to bridge molecular mechanisms and their effects on subjective experience, cognition, and behavior via their effects on the brain’s functional architecture.

Using our computational framework, we found that psychoactive drugs can be understood in terms of contributions from multiple neurotransmitter systems. We also found that anesthetics and psychedelics/cognitive enhancers are largely opposite in terms of their association with neurotransmitters in the cortex, although not without exceptions. The effects of mind-altering drugs are topographically organized along multiple hierarchical gradients of brain function, anatomy, and neurobiology. Last, we found that co-susceptibility to pharmacological perturbations recapitulates co-susceptibility to disorder-induced structural perturbations.

The diverse mapping between drug-induced functional reorganization and neurotransmitters that we recovered (Fig. 5) clearly shows the power of our multivariate approach for detecting both expected and previously unidentified relationships between drugs and neurotransmitters. On one hand, we found only weak alignment between ketamine and NMDA receptors, which it is known to antagonize, and between MDMA and the serotonin 2A receptor, despite this being one of its main known targets. On the other hand, MDMA did align strongly with serotonin and dopamine transporters, which it is known to block. Likewise, noradrenaline transporter blocking is one of the main mechanisms of action of methylphenidate, and we also observed high alignment between the two. We also observed that both the serotonergic psychedelics and the traditional GABAergic anesthetics vary in their alignment with their main molecular targets (5HT-2A and GABA_A_ receptors, respectively). Such multifaceted results can emerge from numerous biological mechanisms. Not only are many of the drugs that we considered known to have varied molecular targets, beyond the primary ones through which they exert their effects; in addition, even when a drug has low affinity for a given receptor when considered in isolation, it may still indirectly engage the corresponding neurotransmitter system through complex downstream effects, which abound in the brain. In addition, the highest alignment with GABA_A_ receptor expression was found for the lowest dose of anesthetics—possibly suggesting that, as the dose increases, secondary and downstream targets may begin to exert more prominent effects, thereby contributing to a more diffuse mapping onto the full set of neurotransmitters. As a data-driven technique designed to identify many-to-many mappings rather than focusing on individual ones (*67*), PLS is especially suitable for detecting such multifaceted effects.

Overall, our analytic approach can contribute to uncovering the large-scale downstream effects of each drug on various neurotransmitter systems in a systematic, data-driven fashion. Although the associations identified by our multivariate mapping are correlational and cannot distinguish agonism from antagonism, they can inform future causal experimentation. For instance, upon finding that a drug’s effects spatially covary with the regional density of a receptor that is not among its known molecular targets in vitro, it may indicate an indirect involvement of this neurotransmitter system. This hypothesis generated by our data-driven method may be then probed causally by assessing whether the drug’s effects on the brain and cognition persist if the receptor in question is blocked.

The present results add another dimension to recent work using a similar multivariate approach to relating gene expression of receptors with subjective reports of psychedelic experiences, which also found widespread involvement of multiple receptors (*38*). In addition, the drugs we considered here have profound effects on the mind after a single acute dose, from cognitive enhancement to hallucinations to the suppression of consciousness together. Such far-reaching effects are accompanied by sometimes marked repercussions on brain function and dynamics: It stands to reason that such widespread reorganization would not leave many neurotransmitter pathways unaffected—even those that are not directly involved in generating the altered state in question.

The broadly opposite characterization of traditional anesthetics and most psychedelics is aligned with their respective effects on the complexity of brain activity and connectivity, which is reduced by GABAergic anesthesia but increased by LSD, ayahuasca, and psychedelic doses of ketamine, as well as other psychedelics (*13*, *17*, *39*, *50*, *80*–*86*). Similarly, psychedelics (including subanesthetic ketamine) and anesthetics were recently shown to exert opposite effects on structure-function coupling: whereas anesthesia increases the dependence of brain activity on the underlying structural network, LSD, psilocybin, and subanesthetic ketamine induce fMRI BOLD signals that are increasingly liberal with respect to the underlying structural network organization (*39*). We found that anesthetic doses of ketamine align more closely with psychedelic (subanesthetic) doses of ketamine than with anesthetics such as propofol and sevoflurane. Although the ketamine-anesthetized volunteers were behaviorally unresponsive, as for the traditional anesthetics, they subsequently reported a wide range of vivid hallucinatory experiences (*87*). Thus, as per ketamine’s characterization as a dissociative anesthetic, in subjective terms, their consciousness was not suppressed but rather profoundly altered in a manner more similar to psychedelics than anesthetics. Therefore, the neurotransmitter signatures of the two levels of ketamine align with the molecular effects and subjective effects, rather than with the behavioral effects.

The main division we observed in terms of neurotransmitters is between receptors and transporters, which displayed opposite associations with drug-induced effects. Specifically pertaining to PLS1, we found that transporters covary with cognitive enhancers and most psychedelics in primary sensory and motor regions, whereas receptors covary with GABAergic anesthetics in transmodal association cortices.

Hierarchical organization of pharmacologically induced functional reorganization stands to reason based on prior evidence: Both psychedelics and anesthetics have been shown to have potent effects on the activity and connectivity of higher-order association cortices, and the default mode network in particular (*13*, *47*, *48*, *54*, *87*–*89*). In addition, serotonergic psychedelics also exert powerful influences on the visual cortex at the other end of the cortical hierarchy (*47*), and, as a result, they have been shown to induce a “flattening” of the principal gradient of FC (*90*).

Having established that the effects of mind-altering drugs are hierarchically organized, the question then becomes: Why should mind-altering drugs exert their effects in such a hierarchically organized fashion? Multiple aspects of neuroanatomy may contribute to the emergence of this spatial pattern, manifested as a difference in pharmacological response between unimodal and transmodal cortices. First, the principal component of variation of receptor expression is itself organized along the brain’s sensory-to-association hierarchical axis (*21*)—and so is, for instance, the distribution of the serotonin 2A receptor, the main direct target of serotonergic psychedelics (*23*). Second, transmodal cortices differ from unimodal cortices in terms of increased excitability (*91*) and a predominance of feedback-efferent connections (*21*): Combined with their higher diversity of receptor expression across layers (*21*), these regions may be especially susceptible to receive and amplify multiple pharmacological influences—another reason why their response will differ from the response of unimodal regions.

Third, unimodal and transmodal cortices also differ in terms of cerebral blood flow. Since ultimately the bloodstream is how drugs reach their regional molecular targets, greater cerebral blood flow in transmodal than in unimodal cortices may be one of the factors shaping a unimodal-transmodal spatial pattern of response to pharmacological intervention, due to the different availability of the drug (although it should be noted that some drugs can also have effects on heart rate and neurovascular coupling). Last, another feature differentiating unimodal from transmodal cortices is that the latter have higher neuron and synapse density (*74*, *92*) and tend to have more numerous, far-reaching, and diversely distributed anatomical connections (*93*), as well as the highest prevalence of synergistic (complementary) interactions with the rest of the brain (*74*). Thus, any effects that are exerted in transmodal regions may be more likely to quickly reverberate throughout the whole cortex, compared with effects that are primarily exerted in the unimodal cortex.

To summarize, we conjecture that the unimodal-transmodal spatial organization of pharmacologically induced changes in FC may be at least in part explained by several relevant differences between the microscale and macroscale architecture of unimodal and transmodal cortices: Transmodal association cortices are especially diverse in their receptor profiles, and rich in some key receptors; in addition to being more susceptible to pharmacological intervention due to higher expression of receptors, blood flow is poised to bring greater amounts of drug to these very cortices, and once these cortices’ activity is perturbed, the perturbation can reverberate widely, thanks to their widespread and diverse connectivity. Together, these factors are likely to impart differences in the way that unimodal and transmodal cortices respond to pharmacological intervention, which becomes reflected in the unimodal-transmodal spatial pattern that we observed. In other words, since unimodal and transmodal cortices differ along dimensions that are relevant for pharmacology, it stands to reason to observe that pharmacological responses exhibit a pattern of unimodal-transmodal spatial differentiation. Of course, the drugs we included were chosen precisely because of their powerful effects on cognition and subjective experience, so it stands to reason that their effects should align with the division between primary and higher-order cortices (which also aligns with the principal component of variation obtained from NeuroSynth term-based meta-analysis). In other words, drugs whose effects on FC are less selective for higher versus lower ends of the cortical hierarchy may simply be less likely to exert mind-altering effects of the kind that we chose to focus on in this work.

More broadly, we found that pairs of regions that are more similar in terms of their susceptibility to pharmacologically induced FC changes are also more similar in their susceptibility to cortical alterations associated with a variety of neuropsychiatric disorders. This observation suggests a broader pattern of both pharmacological (acute) and neuroanatomical (chronic) susceptibility across regions. We speculate that this joint susceptibility may be related to regional relevance for cognitive function: We found that this joint vulnerability can be understood in terms of two multimodal principal gradients of variation over the cortex: one of them resembling the principal gradient of FC (and principal latent variable of neurotransmitter-drug association) and the other anchored in the dorsolateral prefrontal cortex at one end, and ventromedial and temporal cortex at the other. The first, hierarchically organized gradient of association between disorder co-susceptibility and co-susceptibility to pharmacologically induced functional reorganization sheds light on recent evidence, which indicated that the principal gradient of neurotransmitter expression is particularly relevant for predicting a wide spectrum of disease-specific cortical morphology (*65*). Specifically, the results presented here show that this observation extends to the effects of engaging different receptors. This interpretation is further supported by our own evidence that pharmacological perturbations are shaped by neurotransmitter coexpression. Pertaining to the second, ventromedial-dorsolateral gradient of susceptibility, our NeuroSynth contextualization showed that it coincides with meta-analytic maps related to emotion and valence but also to loss, stress, fear, psychosis, and anxiety. We note that the NeuroSynth fMRI database is independent of the ENIGMA datasets of cortical morphometry that contributed to the generation of our joint gradients. Therefore, the relevance of these terms as potential symptoms or precipitating factors for mental illness provides important validation for our second gradient of joint susceptibility. Together, our two joint gradients delineate cognitively relevant dimensions of cortical susceptibility to pharmacological and pathological perturbations. The results reported here open previously unidentified possibilities for data-driven, multivariate mapping between the brain’s high-dimensional neurotransmitter landscape and the effects of potent pharmacological interventions on the brain’s functional architecture. Crucially, neuropsychiatric disorders and candidate pharmacological treatments for them ultimately need to exert their effects on cognition and behavior by influencing brain function. In this light, it is intriguing that susceptibility to disorder-related cortical abnormalities correlates with susceptibility to pharmacological intervention. This observation suggests that regions that are structurally most vulnerable to disease (which presumably in turn shapes their functional architecture) may also be the ones that are most susceptible to rebalancing their functional organization by an appropriate choice of pharmacological intervention. This work represents the necessary first step toward identifying previously undiscovered and perhaps unexpected associations between drugs and neurotransmitters, as well as elucidating the known ones in a data-driven manner.

Our joint mapping of pathological and pharmacological perturbations, in the space of the two principal gradients of co-susceptibility, represents a proof of principle for how future work can build on our approach. For example, although the pharmacological agents that we included were primarily chosen in virtue of their potent mind-altering effects, rather than their relevance for psychiatric treatment, we still identified an intriguing proximity of schizophrenia-related anatomical alterations with the functional effects of ketamine (particularly in terms of alignment with the first joint gradient), reminiscent of the evidence that ketamine can have “psychoto-mimetic” effects (*30*). By considering patterns of functional rather than structural abnormality associated with pathology, future work may leverage our computational framework to identify drugs—or combinations thereof—whose functional effects are best suited to counteract those of a given disorder.

### Limitations and future directions

Although the main strength of our study is our extensive coverage of both neurotransmitters and pharmacological data, it is important to acknowledge that neither is complete: In particular, our sample did, by no means, exhaustively include all mind-altering drugs that have been studied: Prominent additions for future work may include the psychedelic kappa opioid receptor agonist salvinorin A (*94*), the sedative dexmedetomidine, an alpha-2 receptor agonist (*95*)—but also alcohol or caffeine, arguably the two most widely used psychoactive substances. We also acknowledge that, owing to the complexity of these study designs and the challenges of their implementation, the pharmacological datasets included here come from limited samples that have been studied before, and future replication in different datasets with the same drugs (as we have done here for propofol) would also be desirable. In particular, small samples can increase variability and may consequently produce less reliable results; therefore, replicating the present results in larger samples will be an important consideration for future work.

The datasets included here come from different sources and locations and were acquired under a variety of conditions. We endeavored to mitigate scanner and acquisition differences by re-preprocessing all data with the same pipeline, and following uniform denoising procedures, rather than following the various pipelines originally used by each group. Further mitigation of the acquisition differences between datasets should come from our within-subject design in healthy individuals [except for the methylphenidate dataset, which comes from patients with TBI (*6*); although we showed that our results remain qualitatively the same if this dataset is excluded; fig. S7]. Nevertheless, we cannot exclude some residual influence of such differences on our results (e.g., eyes open versus closed; the ayahuasca data were acquired at a lower field strength of 1.5 T; the TRs varied from 1.671 s for modafinil, to 3 s for psilocybin; table S1). In addition, the drug doses used were different across studies, so our comparisons combine together differences in drug type and drug dose, which will need to be further disentangled in future, dedicated studies.

Similar considerations about the differences between datasets apply for the PET data, as discussed in detail in the original publication collecting the PET maps (*23*). Likewise, the coverage of neurotransmitter receptors and transporters, though the most extensive available to date and obtained in vivo rather than postmortem, is far from exhaustive. The same limitation also applies to the ENIGMA disorder data (*75*): Many more disorders, diseases, and conditions exist than the ones considered here. And although the ENIGMA consortium provides datasets from large samples with standardized pipelines, ensuring robust results, the patient populations may exhibit comorbidities and/or be undergoing treatment. In addition, the available maps do not directly reflect changes in tissue volume, but rather the effect size of patient-control statistical comparisons, in terms of only one low-resolution cortical-only parcellation.

In addition to the inevitable limitations of analyzing large-scale datasets from multiple sites, there are also limitations of our analytic framework. Although we report a macroscale spatial association between neurotransmitter expression and pharmacologically induced functional reorganization that is statistically unexpected on the basis of autocorrelation alone, caution is warranted when drawing inferences from statistical results to the underlying biology. We used linear models that assume independence between observations—an assumption that mostly does not hold in the brain, given the possibility of nonlinear effects in how drugs exert their effects on the brain’s intricately connected neurotransmitter systems. To mitigate this limitation, throughout this work, we triangulated toward a robust statistical mapping between neurotransmitters and drugs by combining cross-validation and different conservative null models that account for the spatial dependencies between regions (*44*).

Another limitation is that, due to data availability and well-documented differences in PET radioligand uptake between cortical and subcortical structures (*23*, *96*, *97*), our work was mainly restricted to the cortex—although we did replicate our results when cortex and subcortex were combined. The thalamus, brainstem, and other subcortical structures are prominently involved in mediating cortico-cortical interactions and the effects of psychedelics, anesthetics, and cognitive enhancers (*11*, *13*, *36*, *54*, *95*, *98*–*102*). We expect that future work suitable data for whole-brain coverage (ideally including cerebellum and brainstem) may provide richer insights than the sum of their individual contributions.

More broadly, the other main limitation of this work is its correlational nature: Receptors and drugs were mapped in separate cohorts of individuals, and identifying spatially correlated patterns does not guarantee the causal involvement of the neurotransmitters in question. Likewise, although our pharmacological manipulations induced behavioral and subjective effects (e.g., loss of responsiveness for the anesthetics), the present work did not seek to provide a direct link between drugs, their molecular targets in the brain, and their behavioral effects. Experimental interventions will be required to conclusively demonstrate causal involvement and elucidate the underlying neurobiological pathways from receptors to behavior. However, we emphasize that our results generate empirically testable hypotheses about which neurotransmitters may be involved with the macroscale effects of different drugs on brain function. These hypotheses may be tested experimentally, but also in silico: Whole-brain computational modeling is becoming increasingly prominent as a tool to investigate the causal mechanisms that drive brain activity and organization in healthy and pathological conditions (*103*, *104*). Crucially, the more biologically inspired models (e.g., dynamic mean field) can also be enriched with further information, such as regional myelination (*91*), or the regional distribution of specific receptors and ion channels obtained from PET or transcriptomics (*57*, *58*, *105*), to reflect neurotransmitter influences. This approach may complement experimental manipulations, making it possible to systematically evaluate the causal effects of combinations of different neuromodulators on the brain’s FC.

Here, we mapped the functional chemoarchitecture of the human brain, by relating the regional changes in fMRI FC induced by 10 different mind-altering drugs, and the regional distribution of 19 neurotransmitter receptors and transporters obtained from PET. This work provides a computational framework to characterize how mind-altering pharmacological agents engage the brain’s rich neurotransmitter landscape to exert their effects on brain function. Our analytic workflow could find application across the breadth of human cognitive and clinical neuroscience, with the potential to shed light on alterations of neurotransmission underlying neuropsychiatric conditions, which are known to involve a combination of anatomical and neurochemical imbalances. More broadly, our framework could also find fruitful application for data-driven prediction of the effects of candidate drugs on the brain: The mapping between neurotransmitters and pharmacological effects on brain function offers an indispensable biological lens that can reveal neurotransmitter targets for therapeutic intervention. In summary, we demonstrate that diverse patterns of neurotransmitter expression are variously engaged by an array of potent pharmacological interventions, ultimately manifesting as a large-scale hierarchical axis. Collectively, these results highlight a statistical link between molecular dynamics and drug-induced reorganization of functional architecture.

## MATERIALS AND METHODS

### Experimental design

PLS analysis was used to relate regional neurotransmitter density to pharmacologically induced FC changes in a multivariate fashion. PLS analysis is an unsupervised multivariate statistical technique that decomposes relationships between two datasets (in our case, neurotransmitter density with *n* being the regions and *r* being the neurotransmitters, *X_nxr_*, and drug-induced FC changes, *Y_nxd_*, with *n* being the regions and *d* being the drugs) into orthogonal sets of latent variables with maximum covariance, which are linear combinations of the original data (*67*). In other words, PLS finds components from the predictor variables (100 × 19 matrix of regional neurotransmitter receptor and transporter density scores) that have maximum covariance with the response variables (100 × 15 matrix of regional changes in FC induced by different drugs). The PLS components (i.e., linear combinations of the weighted neurotransmitter density) are ranked by the covariance between predictor and response variables so that the first few PLS components provide a low-dimensional representation of the covariance between the higher dimensional data matrices. Thus, the first PLS component (PLS1) is the linear combination of the weighted neurotransmitter density scores that have a brain expression map that covaries the most with the map of regional FC changes.

This is achieved by *z*-scoring both data matrices column-wise and applying singular value decomposition on the matrix *Y′X*, such that

$${\left(Y^{\prime }X\right)}^{\prime }={USV}^{\prime }$$ (1)

where *U_g×t_* and *V_t×t_* are orthonormal matrices consisting of left and right singular vectors and *S_t×t_* is a diagonal matrix of singular values. The *i*th columns of *U* and *V* constitute a latent variable, and the *i*th singular value in *S* represents the covariance between singular vectors. The *i*th singular value is proportional to the amount of covariance between neurotransmitter density, and drug-induced FC changes captured by the *i*th latent variable, where the effect size can be estimated as the ratio of the squared singular value to the sum of all squared singular values. In the present study, the left singular vectors (that is, the columns of *U*) represent the degree to which each neurotransmitter contributes to the latent variable and demonstrate the extracted association between neurotransmitter density and drug-induced FC changes (neurotransmitter weights). The right singular vectors (that is, the columns of *V*) represent the degree to which the FC changes contribute to the same latent variable (term weights). Positively weighed neurotransmitters covary with positively weighed drug-induced changes, and negatively weighed neurotransmitters covary with negatively weighed drug-induced changes.

Scores at each brain region for each latent variable can be computed by projecting the original data onto the singular vector weights. Positively scored brain regions are regions that demonstrate the covariance between the prevalence of positively weighted neurotransmitters and positively weighted drug-induced effects (and vice versa for negatively scored brain regions). Loadings for each variable were computed as Pearson’s correlation between each individual variable’s regional distribution (neurotransmitter density and drug-induced FC changes) and the PLS analysis–derived neurotransmitter score pattern. Squaring the loading (a correlation) equals the percentage of variance shared between an original variable and the PLS analysis–derived latent variable. Variables with high absolute loadings are highly correlated to the score pattern, indicating a large amount of shared variance between the individual variable and the latent variable. We confirmed that PLS1 explained the largest amount of variance by testing across a range of PLS components (between 1 and 15) and quantifying the relative variance explained by each component.

### Summarizing pharmacological effects on brain function

For each subject at each condition (drug and baseline), the denoised regional blood oxygen level–dependent (BOLD) signals from fMRI (see the Supplementary Materials) were parcellated into 100 cortical regions according to the local-global functional parcellation of Schaefer and colleagues (*64*). The parcellated regional BOLD signals were then correlated pairwise across regions, obtaining a region-by-region matrix of “functional connectivity”; after removing negative-valued edges (i.e., applying an absolute threshold of zero to the FC matrix), the regional weighted degree of FC (weighted degree) was measured for each region. The regional change in FC weighted degree between the drug and no-drug conditions was then quantified for each subject. Last, for each dataset, we computed the mean (across subjects) of the FC weighted degree deltas. Therefore, each pharmacological intervention was summarized as one vector of regional FC deltas (Fig. 1). We also repeated our analysis with alternative parcellation schemes: 114 cortical regions from the Lausanne atlas (*79*); 32 subcortical regions from the Tian atlas (*106*); and a combination of the same 32 subcortical regions, with 200 cortical regions from the Schaefer atlas. For the latter, cortex and subcortex were *z*-scored separately before being combined for the PLS analysis, due to the well-documented differences in PET radioligand uptake between cortical and subcortical structures (*65*, *97*).

### Receptor maps from positron emission tomography

Receptor densities were estimated using PET tracer studies for a total of 19 receptors and transporters, across nine neurotransmitter systems, recently made available by Hansen and colleagues (*23*). These include dopamine (D1, D2, and DAT), noradrenaline (NAT), serotonin (5-HT1A, 5-HT1B, 5-HT2A, 5-HT4, 5-HT6, and 5-HTT), acetylcholine (α4β2, M1, and VAChT), glutamate (mGluR5 and NMDA), GABA (GABA_A_), histamine (H3), cannabinoid (CB1), and opioid (MOR). Volumetric PET images were registered to the MNI-ICBM 152 nonlinear 2009 (version c, asymmetric) template, averaged across participants within each study, then parcellated, and receptors/transporters with more than one mean image of the same tracer (5-HT1b, D2, and VAChT) were combined using a weighted average. See the dedicated article by Hansen *et al*. (*23*) for detailed information about each PET dataset and their respective acquisition and limitations, and see Description of Supplementary References Excel file for a list of studies that provided data pertaining to each receptor and transporter.

### Hierarchical organization

We quantified the spatial similarity of our latent variable scores, with several canonical maps of hierarchical brain organization (“canonical brain hierarchies”) derived from multimodal neuroimaging. We considered the anatomical gradient of intracortical myelination obtained from T1w/T2w MRI ratio (*43*); evolutionary cortical expansion obtained by comparing human and macaque (*71*); the principal component of variation in gene expression from the AHBA transcriptomic database (https://human.brain-map.org/), referred to as “AHBA PC1” (*40*, *44*, *72*); the principal component of variation in task activation from NeuroSynth, an online meta-analytic tool that synthesizes results from more than 15,000 published fMRI studies by searching for high-frequency keywords that are published alongside fMRI voxel coordinates, using the volumetric association test maps (referred to as “NeuroSynth PC1”) (*40*, *44*, *73*); the map of cerebral blood flow (*40*); the principal gradient of variation in FC (*42*); and a recently derived gradient of the regional prevalence of different kinds of information, from redundancy to synergy (*74*).

### Contextualization with NeuroSynth meta-analytic maps

As an alternative avenue to characterize cortical patterns, we quantified their spatial similarity with 123 term-based meta-analytic brain maps from the NeuroSynth database. The probabilistic measure reported by NeuroSynth can be interpreted as a quantitative representation of how regional fluctuations in activity are related to psychological processes. Although more than 1000 terms are reported in NeuroSynth, we focus primarily on cognitive function and therefore limit the terms of interest to cognitive and behavioral terms (*44*, *73*). The resulting 123 terms, range from umbrella terms (attention and emotion) to specific cognitive processes (visual attention and episodic memory), behaviors (eating and sleep), and emotional states (fear and anxiety).

### ENIGMA cortical vulnerability data

Patterns of cortical thickness were collected for the available 11 neurological, neurodevelopmental, and psychiatric disorders from the ENIGMA consortium and the *enigma* toolbox (https://enigma-toolbox.readthedocs.io/en/latest/) (*65*, *75*, *76*): 22q11.2 deletion syndrome, attention-deficit/hyperactivity disorder, autism spectrum disorder, idiopathic generalized epilepsy, right temporal lobe epilepsy, left temporal lobe epilepsy, depression, obsessive-compulsive disorder, schizophrenia, bipolar disorder, and Parkinson’s disease. See Description of Supplementary References Excel file for a list of studies that provided data pertaining to each disease and disorder. The ENIGMA consortium is a data-sharing initiative that relies on standardized image acquisition and processing pipelines, such that disorder maps are comparable (*76*). Structural T_1_-weighted MRI scans acquired at the various contributing sites were segmented using standardized and publicly available ENIGMA imaging protocols. These automated protocols, based on FreeSurfer (version 5.3) segmentations, are fully validated and allow maximal uniformity and comparability across sites. Together, more than 17,000 patients were scanned across the 13 disorders, against almost 22,000 controls. The values for each map are *z*-scored effect sizes (Cohen’s *d*) of cortical thickness in patient populations versus healthy controls. Imaging and processing protocols can be found at http://enigma.ini.usc.edu/protocols/.

For every brain region, we constructed an 11-element vector of disorder abnormality, where each element represents a disorder’s cortical abnormality at the region. For every pair of brain regions, we correlated the abnormality vectors to quantify how similarly two brain regions are affected across disorders. This results in a region-by-region matrix of “disorder co-susceptibility” (*65*).

### Gradients from diffusion map embedding

We used the *BrainSpace* toolbox (*77*) to obtain gradients from diffusion map embedding. A joint network of disease and drug co-susceptibility was obtained from the network fusion procedure of Paquola *et al*. (*78*), through horizontal concatenation of matrices and production of a node-to-node affinity matrix using row-wise normalized angle similarity.

We then used diffusion map embedding, a nonlinear manifold learning technique based on the graph Laplacian (*77*), to obtain a low dimensional representation of the joint drug and disorder susceptibility. A single parameter α controls the influence of the sampling density on the manifold (α = 0, maximal influence; α = 1, no influence). Following extensive previous work using this approach with neuroimaging data (*42*, *77*), we set α = 0.5 to retain the global relations between data points in the embedded space. The ability to combine global and local geometry differentiates diffusion maps from global-only methods, such as principal components analysis and multidimensional scaling (*78*). A small number of components can be identified on the basis of decreasing eigenvalues. The decay of each eigenvector obtained from the diffusion map embedding provides an overall measure of the connectivity between nodes along the axis delineated by its spatial distribution on the brain (gradient).

### Statistical analysis

The statistical significance of the variance explained by each PLS model was tested by permuting the response variables 1000 times while considering the spatial dependency of the data by using spatial autocorrelation-preserving permutation tests, termed spin tests (*68*, *69*). Parcel coordinates were projected onto the spherical surface and then randomly rotated and original parcels were reassigned the value of the closest rotated parcel (10,000 repetitions). The procedure was performed at the parcel resolution rather than the vertex resolution to avoid upsampling the data. In PLS analysis, the spin test is applied to the singular values (or equivalently, the covariance explained) of the latent variables, producing a null distribution of singular values. This is done by applying PLS analysis to the original *X* matrix and a spun *Y* matrix. The spin test embodies the null hypothesis that neurotransmitter density and drug-induced FC changes are spatially correlated with each other only because of inherent spatial autocorrelation. The *P* value is computed as the proportion of null singular values that are greater in magnitude than the empirical singular values. Thus, these *P* values represent the probability that the observed spatial correspondence between neurotransmitter density and drug-induced FC changes could occur by randomly correlating maps with comparable spatial autocorrelation.

As an alternative null model, we also derived a null distribution of singular values by repeating the PLS analysis 1000 times, with a null *Y* matrix obtained by randomly permuting subjects’ drug and no-drug conditions. The *P* values obtained against this null distribution represent the probability that the observed spatial correspondence between neurotransmitter density and drug-induced FC changes could occur by chance even in the absence of drug effects.

Spatial similarity between brain maps was quantified in terms of Spearman’s correlation, and statistical significance was assessed against a spin-based null model with preserved spatial autocorrelation, as described above (*68*, *69*, *107*). Correction for multiple comparisons was implemented using the FDR method of Benjamini and Hochberg (*108*).

We also implemented a distance-dependent cross-validation method (*65*): The correlation between drug scores and neurotransmitter scores was cross-validated by constructing a training set with 75% of brain regions closest in Euclidean distance to a randomly chosen source node, with the testing set comprising the remaining 25% of regions that are farthest away from the training set nodes. The out-of-sample mean is then assessed against a permuted null model (1000 repetitions). This technique attempts to account for the spatial autocorrelation of the brain by testing the fitted model on a held-out set of brain regions that are spatially distant and therefore likely divergent in annotation properties (*44*).

To further account for the potential confound of signal from nongray matter tissue (which can give rise to partial volume effects by contaminating the gray matter signal) (*109*), we used the probability of nongray matter tissue in each region as a regressor. We parcellated an a priori gray matter tissue probability map (*110*) to obtain the prevalence of gray matter in each ROI (mean of the gray matter probability of its constituent voxels). The complement of this map represents the probability of nongray matter tissue in each region, which we used as an estimate of each region’s susceptibility to contamination from nongray matter tissue signal. This probability map was then regressed out of the latent variables, before computing their spatial correlation with cortical hierarchy maps, as a control analysis. Similarly, when correlating matrices, a control analysis was performed by using as regressor a matrix of pairwise similarity between regions’ probability of containing nongray-matter tissue, obtained as the outer product of the *z*-scored vector of nongray matter tissue probability with itself.

When considering multiple cortical hierarchies, we also applied dominance analysis to evaluate their relative contribution to our latent variable scores. Dominance analysis seeks to determine the relative contribution (“dominance”) of each independent variable to the overall fit (adjusted *R^2^*) of the multiple linear regression model (*23*, *111*). This is done by fitting the same regression model on every combination of predictors (2*^p^* − 1 submodels for a model with *p* being the predictors). Total dominance is defined as the average of the relative increase in *R^2^* when adding a single predictor of interest to a submodel, across all 2*^p^* − 1 submodels. The sum of the dominance of all input variables is equal to the total adjusted *R^2^* of the complete model, from which a percentage of relative importance is obtained by partitioning the total effect size accounted for by each predictor. Therefore, unlike other methods of assessing predictor importance, such as methods based on regression coefficients or univariate correlations, dominance analysis accounts for predictor-predictor interactions and is interpretable.

## Supplementary Materials

### This PDF file includes:

Supplementary Methods Figs. S1 to S19 Tables S1 and S2 Legend for Auxiliary datasets file Legend for Supplementary References Excel file

### Other Supplementary Material for this manuscript includes the following:

Auxiliary datasets file

Supplementary References Excel file

## Notes

View/request a protocol for this paper from *Bio-protocol*.
