# Supplementary material for: In vivo mapping of pharmacologically induced functional reorganization onto the human brain’s neurotransmitter landscape
Source: Sci Adv. 2023 Jun 14;9(24):eadf8332. doi: 10.1126/sciadv.adf8332 (PMC10266734; doi:10.1126/sciadv.adf8332)
Supplement: Supplementary file 1 — Supplementary Methods Figs. S1 to S19 Tables S1 and S2 Legend for Auxiliary datasets file Legend for Supplementary References Excel file [file sciadv.adf8332_sm.pdf]

Science Advances
AAAS

Supplementary Materials for

In vivo mapping of pharmacologically induced functional reorganization onto the human brain's neurotransmitter landscape

Andrea I. Luppi *et al.*

Corresponding author: Andrea I. Luppi, al857@cam.ac.uk

Sci. Adv. **9**, eadf8332 (2023)
DOI: 10.1126/sciadv.adf8332

The PDF file includes:

Supplementary Methods
Figs. S1 to S19
Tables S1 and S2
Legend for Auxiliary datasets file
Legend for Supplementary References Excel file

Other Supplementary Materials includes the following:

Auxiliary datasets file
Supplementary References Excel file

Supplementary Methods

Description of datasets

Propofol

Propofol (2,6-diisopropylphenol) is perhaps the most common agent used for intravenous induction and maintenance of general anaesthesia(*24*). One of the chief reasons for its widespread use, both in the operating room and for scientific studies, is propofol's rapid action, which allow for precise titration and therefore greater control over the induction and emergence process. Additionally, propofol has minimal effects on both regional cerebral blood flow(*113*), and the coupling between blood flow and metabolism(*114*), thereby reducing the number of potential confounding effects. Propofol is a potent agonist of inhibitory GABA-A receptors, directly activating them as well as increasing their affinity for agonists(*24*, *25*, *115*), leading to suppressed neuronal activity. Propofol also blocks Na+ channels, inhibiting glutamate release(*28*, *116*) and more broadly it inhibits neurotransmitter release at presynaptic terminals(*117*). There is also some evidence that it may affect the dopaminergic system(*36*, *118*, *119*). Here, we included two independent propofol datasets.

Cambridge University dataset: Recruitment

The Cambridge University ("Cambridge") propofol dataset is described in detail in a previous publication(*55*). Sixteen healthy volunteer subjects were initially recruited for scanning. In addition to the original 16 volunteers, data were acquired for nine additional participants using the same procedures, bringing the total number of participants in this dataset to 25 (11 males, 14 females; mean age 34.7 years, SD = 9.0 years). Ethical approval for these studies was obtained from the Cambridgeshire 2 Regional Ethics Committee, and all subjects gave informed consent to participate in the study. Volunteers were informed of the risks of propofol administration, such as loss of consciousness, respiratory and cardiovascular depression. They were also informed about more minor effects of propofol such as pain on injection, sedation and amnesia. In addition, standard information about intravenous cannulation, blood sampling and MRI scanning was provided.

Cambridge University dataset: Study protocol

Three target plasma levels of propofol were used - no drug (Awake), 0.6 mg/ml (Mild sedation) and 1.2 mg/ml (Moderate sedation). Scanning (rs-fMRI) was acquired at each stage, and also at Recovery; anatomical images were also acquired. The level of sedation was assessed verbally immediately before and after each of the scanning runs. Propofol was administered intravenously as a "target controlled infusion" (plasma concentration mode), using an Alaris PK infusion pump (Carefusion, Basingstoke, UK). A period of 10 min was allowed for equilibration of plasma and effect-site propofol concentrations. Blood samples were drawn towards the end of each titration period and before the plasma target was altered, to assess plasma propofol levels. In total, 6 blood samples were drawn during the study. The mean (SD) measured plasma propofol concentration was 304.8 (141.1) ng/ml during mild sedation, 723.3 (320.5) ng/ml during moderate sedation and 275.8 (75.42) ng/ml during recovery. Mean (SD) total mass of propofol administered was 210.15 (33.17) mg, equivalent to 3.0 (0.47) mg/kg. Two senior anaesthetists were present during scanning sessions and observed the subjects throughout the

study from the MRI control room and on a video link that showed the subject in the scanner. Electrocardiography and pulse oximetry were performed continuously, and measurements of heart rate, non-invasive blood pressure, and oxygen saturation were recorded at regular intervals.

Cambridge University dataset: MRI Data Acquisition

The acquisition procedures are described in detail in the original study(*55*). Briefly, MRI data were acquired on a Siemens Trio 3T scanner (WBIC, Cambridge). For each level of sedation, 150 rs-fMRI volumes (5 min scanning) were acquired. Each functional BOLD volume consisted of 32 interleaved, descending, oblique axial slices, 3 mm thick with interslice gap of 0.75 mm and in-plane resolution of 3 mm, field of view = 192x192 mm, repetition time = 2000 ms, acquisition time = 2 s, time echo = 30 ms, and flip angle 78. T1-weighted structural images at 1 mm isotropic resolution were also acquired in the sagittal plane, using an MPRAGE sequence with TR = 2250 ms, TI = 900 ms, TE = 2.99 ms and flip angle = 9 degrees, for localization purposes. During scanning, volunteers were instructed to close their eyes and think about nothing in particular throughout the acquisition of the resting state BOLD data. Of the 25 healthy subjects, 15 were ultimately retained (7 males, 8 females): 10 were excluded, either because of missing scans (n=2), or due of excessive motion in the scanner (n=8, 5mm maximum motion threshold). For the analyses presented in this paper, we only considered the Awake, Moderate (i.e., loss of behavioural responsiveness) and Recovery resting-state scans.

Western University dataset: Recruitment

The Western University ("Western") propofol data were collected between May and November 2014 at the Robarts Research Institute, Western University, London, Ontario (Canada), and have been published before(*13*, *86*, *120*, *121*). The study received ethical approval from the Health Sciences Research Ethics Board and Psychology Research Ethics Board of Western University (Ontario, Canada). Healthy volunteers (n=19) were recruited (18–40 years; 13 males). Volunteers were right-handed, native English speakers, and had no history of neurological disorders. In accordance with relevant ethical guidelines, each volunteer provided written informed consent, and received monetary compensation for their time. Due to equipment malfunction or physiological impediments to anaesthesia in the scanner, data from n=3 participants (1 male) were excluded from analyses, leaving a total n=16 for analysis(*13*).

Western University dataset: Study protocol

Resting-state fMRI data were acquired at different propofol levels: no sedation (Awake), Deep anaesthesia (corresponding to Ramsay score of 5) and also during post-anaesthetic recovery. As previously reported(*13*), for each condition fMRI acquisition began after two anaesthesiologists and one anaesthesia nurse independently assessed Ramsay level in the scanning room. The anaesthesiologists and the anaesthesia nurse could not be blinded to experimental condition, since part of their role involved determining the participants' level of anaesthesia. Note that the Ramsay score is designed for critical care patients, and therefore participants did not receive a score during the Awake condition before propofol administration: rather, they were required to be fully awake, alert and communicating appropriately. To provide a further, independent evaluation of participants' level of responsiveness, they were asked to perform two tasks: a test of verbal memory recall, and a computer-based auditory target-detection task. Wakefulness was also monitored using an infrared camera placed inside the scanner.

Propofol was administered intravenously using an AS50 auto syringe infusion pump (Baxter Healthcare, Singapore); an effect-site/plasma steering algorithm combined with the computer-controlled infusion pump was used to achieve step-wise sedation increments, followed by manual adjustments as required to reach the desired target concentrations of propofol according to the TIVA Trainer (European Society for Intravenous Aneaesthesia, eurosiva.eu) pharmacokinetic simulation program. This software also specified the blood concentrations of propofol, following the Marsh 3-compartment model, which were used as targets for the pharmacokinetic model providing target-controlled infusion. After an initial propofol target effect-site concentration of 0.6 μg mL^{-1}, concentration was gradually increased by increments of 0.3 μg mL^{1}, and Ramsay score was assessed after each increment: a further increment occurred if the Ramsay score was lower than 5. The mean estimated effect-site and plasma propofol concentrations were kept stable by the pharmacokinetic model delivered via the TIVA Trainer infusion pump. Ramsay level 5 was achieved when participants stopped responding to verbal commands, were unable to engage in conversation, and were rousable only to physical stimulation. Once both anaesthesiologists and the anaesthesia nurse all agreed that Ramsay sedation level 5 had been reached, and participants stopped responding to both tasks, data acquisition was initiated. The mean estimated effect-site propofol concentration was 2.48 (1.82- 3.14) μg mL^{-1}, and the mean estimated plasma propofol concentration was 2.68 (1.92- 3.44) μg mL^{-1}. Mean total mass of propofol administered was 486.58 (373.30- 599.86) mg. These values of variability are typical for the pharmacokinetics and pharmacodynamics of propofol. Oxygen was titrated to maintain SpO2 above 96%.

At Ramsay 5 level, participants remained capable of spontaneous cardiovascular function and ventilation. However, the sedation procedure did not take place in a hospital setting; therefore, intubation during scanning could not be used to ensure airway security during scanning. Consequently, although two anaesthesiologists closely monitored each participant, scanner time was minimised to ensure return to normal breathing following deep sedation. No state changes or movement were noted during the deep sedation scanning for any of the participants included in the study(*13*). Propofol was discontinued following the deep anaesthesia scan, and participants reached level 2 of the Ramsey scale approximately 11 minutes afterwards, as indicated by clear and rapid responses to verbal commands. This corresponds to the "recovery" period.

As previously reported(*13*), once in the scanner participants were instructed to relax with closed eyes, without falling asleep. Resting-state functional MRI in the absence of any tasks was acquired for 8 minutes for each participant. A further scan was also acquired during auditory presentation of a plot-driven story through headphones (5-minute long). Participants were instructed to listen while keeping their eyes closed. The present analysis focuses on the resting-state data only; the story scan data have been published separately(*122*) and will not be discussed further here.

Western University dataset: MRI Data Acquisition

As previously reported(*13*), MRI scanning was performed using a 3-Tesla Siemens Tim Trio scanner (32-channel coil), and 256 functional volumes (echo-planar images, EPI) were collected from each participant, with the following parameters: slices = 33, with 25% inter-slice gap; resolution = 3mm isotropic; TR = 2000ms; TE = 30ms; flip angle = 75 degrees; matrix size = 64x64. The order of acquisition was interleaved, bottom-up. Anatomical scanning was also performed, acquiring a high-resolution T1- weighted volume (32-channel coil, 1mm isotropic

voxel size) with a 3D MPRAGE sequence, using the following parameters: TA = 5min, TE = 4.25ms, 240x256 matrix size, 9 degrees flip angle(*13*).

Sevoflurane

Sevoflurane is an inhalational anaesthetic: specifically, a halogenated ether. Although its exact molecular mechanisms of action are yet to be fully elucidated, in vivo and in vitro evidence indicates that it acts primarily via GABA-A receptors(*26*, *27*, *123–126*), but also interacts with NMDA, AMPA(*27*, *127*, *128*) and nicotinic ACh receptors(*129*, *130*). Additionally, electrophysiologic investigation suggests possible affinity for as well as Na+, K+ and hyperpolarization-activated cyclic nucleotide-gated (HCN) channels(*131*).

Sevoflurane dataset: Recruitment

The data included here have been published before (*54*, *132–134*), and we refer the reader to the original publication for details(*54*). The ethics committee of the medical school of the Technische Universität München (München, Germany) approved the current study, which was conducted in accordance with the Declaration of Helsinki. Written informed consent was obtained from volunteers at least 48 h before the study session. Twenty healthy adult men (20 to 36 years of age; mean, 26 years) were recruited through campus notices and personal contact, and compensated for their participation in the study.

Before inclusion in the study, detailed information was provided about the protocol and risks, and medical history was reviewed to assess any previous neurologic or psychiatric disorder. A focused physical examination was performed, and a resting electrocardiogram was recorded. Further exclusion criteria were the following: physical status other than American Society of Anesthesiologists physical status I, chronic intake of medication or drugs, hardness of hearing or deafness, absence of fluency in German, known or suspected disposition to malignant hyperthermia, acute hepatic porphyria, history of halothane hepatitis, obesity with a body mass index more than 30 kg/m2, gastrointestinal disorders with a disposition for gastroesophageal regurgitation, known or suspected difficult airway, and presence of metal implants. Data acquisition took place between June and December 2013.

Sevoflurane dataset: Study protocol

Sevoflurane concentrations were chosen so that subjects tolerated artificial ventilation (reached at 2.0 vol%) and that burst-suppression (BS) was reached in all participants (around 4.4 vol%). To make group comparisons feasible, an intermediate concentration of 3.0 vol% was also used. In the MRI scanner, volunteers were in a resting state with eyes closed for 700s. Since EEG data were simultaneously acquired during MRI scanning (*54*) (though they are not analysed in the present study), visual online inspection of the EEG was used to verify that participants did not fall asleep during the pre-anaesthesia baseline scan. Sevoflurane mixed with oxygen was administered via a tight-fitting facemask using an fMRI-compatible anaesthesia machine (Fabius Tiro, Dräger, Germany). Standard American Society of Anesthesiologists monitoring was performed: concentrations of sevoflurane, oxygen and carbon dioxide, were monitored using a cardiorespiratory monitor (DatexaS/3, General electric, USA). After administering an end-tidal sevoflurane concentration (etSev) of 0.4 vol% for 5 min, sevoflurane concentration was increased in a stepwise fashion by 0.2 vol% every 3 min until the participant became unconscious, as judged by the loss of responsiveness (LOR) to the repeatedly spoken command

“squeeze my hand” two consecutive times. Sevoflurane concentration was then increased to reach an end-tidal concentration of approximately 3 vol%. When clinically indicated, ventilation was managed by the physician and a laryngeal mask suitable for fMRI (I-gel, Intersurgical, United Kingdom) was inserted. The fraction of inspired oxygen was then set at 0.8, and mechanical ventilation was adjusted to maintain end-tidal carbon dioxide at steady concentrations of 33 ± 1.71 mmHg during BS, 34 ± 1.12 mmHg during 3 vol%, and 33 ± 1.49 mmHg during 2 vol% (throughout this article, mean ± SD). Norepinephrine was given by continuous infusion (0.1 ± 0.01 $\mu g \cdot kg^{-1} \cdot min^{-1}$) through an intravenous catheter in a vein on the dorsum of the hand, to maintain the mean arterial blood pressure close to baseline values (baseline, 96 ± 9.36 mmHg; BS, 88 ± 7.55 mmHg; 3 vol%, 88 ± 8.4 mmHg; 2 vol%, 89 ± 9.37 mmHg; follow-up, 98 ± 9.41 mmHg). After insertion of the laryngeal mask airway, sevoflurane concentration was gradually increased until the EEG showed burst-suppression with suppression periods of at least 1,000 ms and about 50% suppression of electrical activity (reached at 4.34 ± 0.22 vol%), which is characteristic of deep anaesthesia. At that point, another 700s of electroencephalogram and fMRI was recorded. Further 700s of data were acquired at steady end-tidal sevoflurane concentrations of 3 and 2 vol%, respectively, each after an equilibration time of 15 min. In a final step, etSev was reduced to two times the concentration at LOR. However, most of the subjects moved or did not tolerate the laryngeal mask any more under this condition: therefore, this stage was not included in the analysis(*54*).

Sevoflurane administration was then terminated, and the scanner table was slid out of the MRI scanner to monitor post-anaesthetic recovery. The volunteer was manually ventilated until spontaneous ventilation returned. The laryngeal mask was removed as soon as the patient opened his mouth on command. The physician regularly asked the volunteer to squeeze their hand: recovery of responsiveness was noted to occur as soon as the command was followed. Fifteen minutes after the time of recovery of responsiveness, the Brice interview was administered to assess for awareness during sevoflurane exposure; the interview was repeated on the phone the next day. After a total of 45 min of recovery time, another resting-state combined fMRI-EEG scan was acquired (with eyes closed, as for the baseline scan). When participants were alert, oriented, cooperative, and physiologically stable, they were taken home by a family member or a friend appointed in advance.

Sevoflurane dataset: MRI Data Acquisition

Although the original study acquired both functional MRI (fMRI) and electroencephalographic (EEG) data, in the present work we only considered the fMRI data. Data acquisition was carried out on a 3-Tesla magnetic resonance imaging scanner (Achieva Quasar Dual 3.0T 16CH, The Netherlands) with an eight-channel, phased-array head coil. The data were collected using a gradient echo planar imaging sequence (echo time = 30 ms, repetition time (TR) = 1.838 s, flip angle = 75°, field of view = 220 × 220 mm^2, matrix = 72 × 72, 32 slices, slice thickness = 3 mm, and 1 mm interslice gap; 700-s acquisition time, resulting in 350 functional volumes). The anatomical scan was acquired before the functional scan using a T1-weighted MPRAGE sequence with 240 × 240 × 170 voxels (1×1×1 mm voxel size) covering the whole brain. A total of 16 volunteers completed the full protocol and were included in our analyses; one subject was excluded due to high motion, leaving N=15 for analysis. Here, we used fMRI data from the Awake, 3% vol, and Recovery scans.

Ketamine

Ketamine is a multi-faceted drug, in terms of both neurophysiology and how it affects subjective experience. Depending on dosage, it can act as a "dissociative" anaesthetic (high dose)(*16*, *18*, *88*, *135*) or as an "atypical psychedelic" (at sub-anaesthetic dose)(*29*, *30*, *136*–*138*). At small doses, it has also found recent use as a fast-acting antidepressant(*139*, *140*). Both the anaesthetic and psychedelic effects of ketamine are in some respect unusual; unlike widely used anaesthetics like propofol and sevoflurane, ketamine does not exert its anaeshtetic function through agonism of GABA receptors, nor does it recruit sleep-promoting hypothalamic nuclei, which it appears to suppress instead(*18*, *137*). Likewise, although ketamine does induce psychedelic-like symptoms such as perceptual distortions, vivid imagery and hallucinations, like classic psychedelics, it also induces prominent dissociative symptoms of disembodiment (*52*, *137*, *141*–*143*). Its psychedelic action is not mediated by the serotonin 2A receptor, on which classic psychedelics operate(*32*, *34*): although its precise mechanisms of action are yet to be fully elucidated, ketamine appears to be primarily an antagonist of NMDA and HCN1 receptors; however, evidence suggests that cholinergic, aminergic, and opioid systems may also play modulatory roles(*31*, *35*, *138*).

<u>Psychedelic ketamine dataset: Recruitment</u>

The sub-anaesthetic ("psychedelic") ketamine data included in this study have been published before(*52*), and we refer the reader to the original publication for details. Briefly, a total of 21 participants (10 males; mean age 28.7 years, SD = 3.2 years) were recruited via advertisements placed throughout central Cambridge, UK(*52*). All participants underwent a screening interview in which they were asked whether they had previously been diagnosed or treated for any mental health problems and whether they had ever taken any psychotropic medications. Participants reporting a personal history of any mental health problems or a history of any treatment were excluded from the study. All participants were right-handed, were free of current of previous psychiatric or neurological disorder or substance abuse problems, and had no history of cardiovascular illness or family history of psychiatric disorder/substance abuse. The study was approved by the Cambridge Local Research and Ethics Committee, and all participants provided written informed consent in accordance with ethics committee guidelines.

<u>Psychedelic ketamine dataset: Study protocol</u>

Participants were scanned (resting-state functional MRI and anatomical T1) on two occasions, separated by at least 1 week. On one occasion, they received a continuous computer-controlled intravenous infusion of a racemic ketamine solution (2 mg/ml) until a targeted plasma concentration of 100 ng/ml was reached. This concentration was sustained throughout the protocol. A saline infusion was administered on the other occasion. Infusion order was randomly counterbalanced across participants. The infusion was performed and monitored by a trained anaesthetist (R.A.) who was unblinded for safety reasons, but who otherwise had minimal contact with participants. At all other times, participants were supervised by investigators blinded to the infusion protocol. The participants remained blinded until both assessments were completed. Bilateral intravenous catheters were inserted into volunteers' forearms, one for infusion, and the other for serial blood sampling. A validated and previously implemented(*144*) three-compartment pharmacokinetic model was used to achieve a constant plasma concentration of 100 ng/ml using a computerized pump (Graseby 3500, Graseby Medical, UK). The infusion continued for 15 min to allow stabilization of plasma levels. Blood samples were drawn before and after the resting fMRI scan and then placed on ice. Plasma was obtained by centrifugation

and stored at −70 °C. Plasma ketamine concentrations were measured by gas chromatography–mass spectrometry.

Psychedelic ketamine dataset: MRI Data Acquisition

All MRI and assessment procedures were identical across assessment occasions. Scanning was performed using a 3.0 T MRI scanner (Siemens Magnetom, Trio Tim, Erlangen, Germany) equipped with a 12-channel array coil located at the Wolfson Brain Imaging Centre, Addenbrooke's Hospital, Cambridge, UK. T2*-weighted echo-planar images were acquired under eyes-closed resting-state conditions. Participants were instructed to close their eyes and let the minds wander without going to sleep. Subsequent participant debriefing ensured that no participants fell asleep during the scan. Imaging parameters were: 3x3x3.75mm voxel size, with a time-to-repetition (TR) of 2000 ms, time-to-echo (TE) of 30 ms, flip angle of 781 in 64x64 matrix size, and 240mm field of view (FOV). A total of 300 volumes comprising 32 slices each were obtained. In addition, high- resolution anatomical T1 images were acquired using a three-dimensional magnetic-prepared rapid gradient echo (MPPRAGE) sequence. In all, 176 contiguous sagittal slices of 1.0mm thickness using a TR of 2300 ms, TE of 2.98 ms, flip angle of 91, and a FOV of 256mm in 240x256 matrix were acquired with a voxel size of 1.0mm^3. One participant was excluded due to excessive movement, resulting in a final sample of N=20 subjects.

Anaesthetic ketamine dataset: Recruitment

The anaesthetic ketamine data used here have been published before (*88*), and we refer the reader to the original publication for details. The original study was approved by approval by the ethics committee of the Medical school of the university of Liege (University Hospital, Liege, Belgium), registered at eudract 2010-023016-13. 14 right-handed volunteers were recruited via advertisements in an Internet forum (5 women; median age [range], 25 [19 to 31] years). Each participant provided written informed consent to participation, and underwent medical interview and physical examination before their participation.

Anaesthetic ketamine dataset: Study protocol

The volunteers were requested to fast for at least 6 h from solids and 2 h from liquids before the experimental session. After structural MR image acquisition, subjects were removed from the MRI scanner, and 64 electroencephalogram (EEG) scalp electrodes were placed to allow for simultaneous EEG-fMRI. Here, we only focus on the functional MRI data.

An 18-gauge intravenous catheter (BD Insyte-W; Becton Dickinson Infusion Therapy Systems inc., USA) was then placed into a vein of the left forearm and infused using normal saline at a rate of 20 ml/h. The intravenous line served for ketamine infusion and eventual administration of rescue medications. a 20-gauge arterial catheter (Arrow International Inc., USA) was also placed into the left radial artery, under strict sterile conditions and after performing local anesthesia with 3 ml of 1% lidocaine. This catheter was equipped with a monitoring set (TruWave, Edwards Lifesciences, Dominican Republic) and served for arterial blood sampling and gas analysis. standard MRI compatible anaesthesia monitoring (Magnitude 3150M; Invivo Research, inc., USA) was also placed to allow continuous monitoring and recording of the electrocardiogram, heart rate, blood pressure, pulse oxymetry (spO2), and breathing frequency throughout the scanning and recovery periods. Through a loosely fitting

plastic facemask, additional oxygen at a rate of 5 l/min was provided to volunteers, whose breathing always remained spontaneous. One certified anaesthesiologist and one neurologist were present throughout the experiment. After setting all needed equipment and monitoring, the volunteers were comfortably installed in the MRI tray. The most comfortable supine position attainable was sought to avoid painful stimulation related to position. all volunteers wore earplugs to attenuate noise and earphones to allow communication with investigators; one investigator remained in the MRI scan room at all times.

Ketamine was administered using a computer-controlled intravenous infusion device composed of a separate laptop computer. A 50-ml syringe was filled with normal saline containing racemic ketamine (Ketalar, Pfizer Ltd., Turkey) at a concentration of 10mg/ml. The pharmacokinetic model used to drive the pump was the domino model, which has been demonstrated to have acceptable predictive performance. This system commands infusion pump rates to allow targeting precise effect-site and plasma concentrations of ketamine, based on several biometric parameters. For each change in ketamine concentration, a 5-min equilibration period was allowed after reaching the target, to permit equilibration of ketamine concentration between body compartments. The depth of sedation was assessed using the Ramsay Scale and the University of Michigan Sedation Scale. Each evaluation took place immediately before and after each fMRi data acquisition sequence. Volunteers were asked to strongly squeeze the hand of the investigator, and the command was repeated twice. For that purpose, and for close watch of the volunteer, an investigator continuously stayed inside the MRI room.

A first fMRI data acquisition was performed in the absence of any infusion of ketamine. Ketamine infusion was then started, and its target concentration was increased by steps of 0.5 μg/ml until a level of sedation corresponding to RS 3 to 4 or UMSS 1 to 2 was reached (light sedation, S1). After the 5-min equilibration period, a novel sequence of data acquisition occurred, consisting of the same sequence of events as during W1. Ketamine target concentration was then further increased by steps of 0.5 μg/ml until RS 5 to 6 or UMSS 4 (deep sedation [s2]), and the same sequence of data acquisition was again performed. Because ketamine has a long elimination half-life and to limit time spent in the fMRI scanner for the volunteer, the temporal order of those clinical states was not randomized. For the same reason, a recovery experimental condition could not be achieved. After those acquisitions, the infusion of ketamine was stopped, and the subject was removed from the fMRI scanner to allow for comfortable recovery. The presence of dreaming during ketamine infusion was checked through a phone call at distance from the experimental session. Here, we only consider the awake and deep sedation scans.

<u>Anaesthetic ketamine dataset: MRI Data Acquisition</u>

MRI data were acquired on a 3T Siemens Allegra scanner (Siemens AG, Germany; Echo Planar Imaging sequence using 32 slices; repetition time = 2,460 ms; echo time = 40 ms; field of view = 220 mm; voxel size = 3.45 × 3.45× 3 mm; and matrix size = 64 × 64× 32). 300 functional volumes were acquired for each volunteer in each condition. A high-resolution structural T1 image was acquired in each volunteer at the beginning of the whole experiment for coregistration to the functional data.

A total of 6 participants had to be excluded from the study and further data analysis because of excessive agitation and movements (5 subjects) or voluntary withdrawal (1 subject), leaving 8 for analysis(*88*).

LSD

LSD (lysergic acid diethylamide) is perhaps the best-known among classic psychedelics, inducing a powerful state of altered consciousness with subjective experiences including hallucinations and "ego dissolution"(*32*, *34*, *145*). Substantial work in humans and animals has demonstrated that LSD influences neuromodulation, having affinity for multiple receptors, primarily serotonergic (5-HT2A, 5-HT1A/B, 5-HT6, 5-HT7) and dopaminergic (D1 and D2 receptors)(*32*, *37*, *145–147*).

The main neural and subjective effects of LSD originate from its agonism of the 5-HT2A receptor: both effects are abolished by pre-treatment with the non-selective 5HT2 antagonist ketanserin, which has highest affinity for the 5HT2A receptor(*101*, *148*). In humans, functional connectivity under LSD shows significant correspondence with the spatial distribution of the 5HT2A receptor(*149*). Providing evidence for a mechanistic role, both PET maps and transcriptomic maps of the 5HT2A receptor (but not other serotonin receptors) have been shown to improve the ability of computational models to recapitulate the effects of LSD on brain activity and connectivity, as measured by fMRI(*57*, *58*, *150*). Therefore, pharmacological and in silico evidence converge towards the central role of the 5HT2A receptor for LSD's ability to alter consciousness and its neural underpinnings – although other receptors have also been shown to play an auxiliary role(*37*).

LSD dataset: Recruitment

The LSD data employed here have been extensively published, and we refer to the original publication for details(*47*). Briefly, collection of these data(*47*) was approved by the National Research Ethics Service Committee London–West London and was conducted in accordance with the revised declaration of Helsinki (2000), the International Committee on Harmonization Good Clinical Practice guidelines and National Health Service Research Governance Framework. Imperial College London sponsored the research, which was conducted under a Home Office license for research with schedule 1 drugs. All participants were recruited via word of mouth and provided written informed consent to participate after study briefing and screening for physical and mental health. The screening for physical health included electrocardiogram (ECG), routine blood tests, and urine test for recent drug use and pregnancy. A psychiatric interview was conducted and participants provided full disclosure of their drug use history. Key exclusion criteria included: < 21 years of age, personal history of diagnosed psychiatric illness, immediate family history of a psychotic disorder, an absence of previous experience with a classic psychedelic drug (e.g. LSD, mescaline, psilocybin/magic mushrooms or DMT/ayahuasca), any psychedelic drug use within 6 weeks of the first scanning day, pregnancy, problematic alcohol use (i.e. > 40 units consumed per week), or a medically significant condition rendering the volunteer unsuitable for the study. Twenty healthy volunteers with previous experience using psychedelic drugs were scanned.

LSD dataset: Study protocol

Volunteers underwent two scans, 14 days apart. On one day they were given a placebo (10-mL saline) and the other they were given an active dose of LSD (75 μg of LSD in 10-mL saline). The order of the conditions was balanced across participants, and participants were blind to this order but the researchers were not. Participants carried out VAS-style ratings via button-press

and a digital display screen presented after each scan, and the 11-factor altered states of conscious- ness (ASC) questionnaire was completed at the end of each dosing day(*47*). All participants reported marked alterations of consciousness under LSD.

The data acquisition protocols were described in detail in the original publication (*47*), so we will only describe them in brief here. The infusion (drug/placebo) was administered over 2 min and occurred 115 min before the resting-state scans were initiated. After infusion, subjects had a brief acclimation period in a mock MRI scanner to prepare them for the experience of being in the real machine. ASL and BOLD scanning consisted of three seven-minute eyes closed resting state scans. The ASL data were not analysed for this study, and will not be discussed further.

LSD dataset: MRI Data Acquisition

The first and third scans were eyes-closed, resting state without stimulation, while the second scan involved listening to music; however, this scan was not used in this analysis. The precise length of each of the two BOLD scans included here was 7:20 minutes. For the present analysis, these two scans were concatenated together in time. Imaging was performed on a 3T GE HDx system. High-resolution anatomical images were acquired with 3D fast spoiled gradient echo scans in an axial orientation, with field of view = 256x256x192 and matrix = 256x256x129 to yield 1mm isotropic voxel resolution. TR/TE = 7.9/3.0ms; inversion time = 450ms; flip angle = 20. BOLD-weighted fMRI data were acquired using a gradient echo planer imaging sequence, TR/TE = 2000/35ms, FoV = 220mm, 64x64 acquisition matrix, parallel acceleration factor = 2, 90 flip angle. Thirty five oblique axial slices were acquired in an interleaved fashion, each 3.4mm thick with zero slice gap (3.4mm isotropic voxels). One subject aborted the experiment due to anxiety and four others were excluded for excessive motion (measured in terms of frame-wise displacement), leaving 15 subjects for analysis (11 males, 4 females; mean age 30.5 years, SD = 8.0 years)(*47*).

Psilocybin

Psilocybin (4-phosphoryloxy-N,N-dimethyltryptamine), a prodrug of psilocin (4-OH-N,N-dimethyltrayptamine), is a classic psychedelic, the active compound of “magic mushrooms” of the *Psilocybe* family. Although its psychedelic effects are exerted via agonism of the serotonin 2A receptor(*11*, *151–154*), psilocin also has demonstrated affinity for additional receptors, in particular serotonin 1A and 2C receptors(*155*).

Psilocybin dataset: Recruitment

Data acquisition for this dataset is described in detail previously(*48*), and will only be summarised here. Volunteers were at least 21 years of age, with no personal or family history of a major psychiatric disorder, no substance dependence, no cardiovascular disease, and no history of adverse response to a psychedelic drug yielded datasets for nine participants. All subjects had used psilocybin at least once before, but not within 6 weeks of the study. The study was approved by a National Health Service research ethics committee and all participants gave informed consent to participate in the study.

Psilocybin dataset: Study protocol

In brief, fifteen health volunteers underwent two MRI scanning sessions at least 14 days apart. In each session, subjects were injected. with either psilocybin (2 mg dissolved in 10 mL of

saline, 60-s intravenous injection) or a placebo (10 mL of saline, 60-s i.v. injection) in a counterbalanced design. The infusions began exactly 6 min after the start of the 12-min fMRI scans and lasted 60s. The subjective effects of psilocybin were felt almost immediately after injection and sustained for the remainder of the scanning session. At the end of each session, subjects were asked to rate the overall intensity of their subjective experience under the drug (or placebo) and to comment on their wakefulness level throughout the scan. As expected, all participants rated the subjective effects of psilocybin (mean intensity = 6.9/10 ± 2.6) as much stronger than placebo (mean intensity = 0.4/10 ± 0.6) and none of the subjects reported falling asleep during either scanning session. The 5 minutes of post-infusion data were used for the present analysis.

Psilocybin dataset: MRI Data Acquisition

Neuroimaging data were acquired using a 3T GE HDx MRI system. Anatomical scans were performed before each functional scan and thus prior to administering either the drug or placebo. Structural scans were collected using a 3D fast spoiled gradient echo scans in an axial orientation, with field of view = 256 × 256 × 192 and matrix = 256 × 256 × 192 to yield 1 mm isotropic voxel resolution (repetition time/echo time TR/TE = 7.9/3.0 ms; inversion time = 450 ms; flip angle = 20). BOLD-weighted fMRI data were acquired at 3T using a gradient echo EPI sequence, TR/TE 3000/35 ms, field-of-view = 192 mm, 64 × 64 acquisition matrix, parallel acceleration factor = 2, 90° flip angle. Fifty-three oblique axial slices were acquired in an interleaved fashion, each 3 mm thick with zero slice gap (3 × 3 × 3-mm voxels). Following the same exclusion criteria for motion described above for the LSD dataset, N=9 subjects were kept for analysis (seven men; age, 32 ± 8.9 SD years of age).

DMT

The tryptamine N,N-Dimethyltryptamine (DMT) is a fast-acting mind-altering drug, capable of inducing an immersive state of altered consciousness, with vivid and detailed visual hallucinations (*156*, *157*). It is found endogenously in trace amounts in the human body(*32*, *158*, *159*), but it is orally inert and therefore primarily studied as intravenous injection, whereupon its effects have very rapid onset (2-5 minutes) and offset, effectively fading within 30 minutes of administration(*160–163*). Pharmacologically, DMT binds to sigma-1 and serotonin (particularly 2A and 2C) receptors, but also dopamine D1 and alpha-adrenergic receptors(*164–167*).

DMT dataset: Recruitment

The original DMT study (*49*) was approved by the National Research Ethics (NRES) Committee London – Brent and the Health Research Authority and was conducted under the guidelines of the revised Declaration of Helsinki (2000), the International Committee on Harmonisation Good Clinical Practices guidelines, and the National Health Service Research Governance Framework. Imperial College London sponsored the research, which was conducted under a Home Office license for research with Schedule 1 drugs. An initial visit was focused on assessing physical and mental health to ensure suitability, and participants provided written informed consent. In total, 20 participants completed all study visits (7 female, mean age = 33.5 years, SD = 7.9).

DMT dataset: Study protocol

This was a single-blind, placebo-controlled, counter-balanced design. Volunteers participated in two testing days, 2 weeks apart. On each testing day, volunteers (after testing for drugs of abuse) were involved in 2 separate scanning sessions. In this initial session (task-free) they received intravenous (IV) administration of either placebo (saline) or DMT (in fumarate form) in a counter-balanced order (half of the participants received placebo and the other half received DMT). This first session always consisted of continuous resting-state scans which lasted 28 minutes with DMT/placebo administered at the end of the 8th minute and scanning was over 20 minutes after injection. Participants lay in the scanner with their eyes closed (an eye mask was used to prevent eyes-opening). Following the scanning procedure, participants were interviewed and completed questionnaires designed to assess the subjective effects experienced during the scan. Here, we focused on the 8 minutes post-infusion, corresponding to peak DMT experience.

DMT dataset: MRI Data Acquisition

MR images were acquired in a 3T MR scanner (Siemens Magnetom Verio syngo MR B17) using a 12-channel head coil for compatibility with EEG acquisition. Functional imaging was performed using a T2*-weighted BOLD sensitive gradient echo planar imaging sequence (repetition time (TR) = 2000ms, echo time (TE) = 30ms, acquisition time (TA) = 28.06 mins, flip angle (FA) = 80°, voxel size = 3.0 x 3.0 x 3.0mm3, 35 slices, interslice distance = 0mm. Whole-brain T1-weighted structural images were also acquired. EEG was also acquired, but here we only focus on the fMRI data. Seven out of 20 participants were discarded from analyses due to excessive movement, or only completing one session, leaving 13 subjects for analysis.

Ayahuasca

The Amazonian beverage ayahuasca is typically used in shamanic religious rituals, where it is obtained as a tea made from two plants: *Psychotria viridis* and *Banisteriopsis caapi*. *Psychotria viridis* contains DMT, which binds to sigma-1 and serotonin (particularly 2A) receptors(*164*, *165*). *Banisteriopsis caapi* contains beta-carboline alkaloids, notably harmine, tetrahydroharmine (THH), and harmaline. As potent monoamine oxidase inhibitors (MAOI), harmine and harmaline prevent the degradation of DMT by liver MAO that would otherwise render it orally inert; additionally, they also increase levels of monoamine neurotransmitters; and THH acts as a mild selective serotonin reuptake inhibitor and a weak MAOI(*168–170*). As for other classic psychedelics, engagement of the 5-HT2A receptor appears to be a necessary condition for the brain and subjective effects of ayahuasca to manifest (*171*).

Ayahuasca dataset: Recruitment

The ayahuasca data that we used have been published before(*50*, *172*), and we refer the reader to the original publication for details. Briefly, data were obtained from 9 healthy right-handed adult volunteers (mean age 31.3, from 24 to 47 years), all who were experienced users of Ayahuasca with at least 5 years use (twice a month) and at least 8 years of formal education. The experimental procedure was approved by the Ethics and Research Committee of the University of São Paulo at Ribeirão Preto (process number 14672/2006). Written informed consent was obtained from all volunteers, who belonged to the Santo Daime religious organisation. All experimental procedures were performed in accordance with the relevant guidelines and regulations. Volunteers were not under medication for at least 3 months prior to the scanning session and were abstinent from caffeine, nicotine and alcohol prior to the acquisition. They had

no history of neurological or psychiatric disorders, as assessed by DSM-IV structured interview71. Subjects ingested 120–200 mL (2.2 mL/kg of body weight) of Ayahuasca known to contain 0.8 mg/mL of DMT and 0.21 mg/mL of harmine. Harmaline was not detected via the chromatography analysis, at the threshold of 0.02 mg/mL7.

Ayahuasca dataset: Study protocol

Volunteers underwent two distinct fMRI scanning sessions: (i) before and (ii) 40 minutes after Ayahuasca intake, when the subjective effects become noticeable (the volunteers drank 2.2 mL/kg of body weight and the Ayahuasca contained 0.8 mg/mL of DMT and 0.21 mg/mL of harmine). In both cases, participants were instructed to close their eyes and remain awake and at rest, without performing any task.

Ayahuasca dataset: MRI Data Acquisition

The fMRI images were obtained in a 1.5 T scanner (Siemens, Magneton Vision), using an EPI-BOLD like sequence comprising 150 volumes, with the following parameters: TR = 1700 ms; TE = 66 ms; FOV = 220 mm; matrix 64 ×64; voxel dimensions of 1.72 mm × 1.72 mm × 1.72 mm. Whole brain high resolution T1-weighted images were also acquired (156 contiguous sagittal slices) using a multiplanar reconstructed gradient-echo sequence, with the following parameters: TR = 9.7 ms; TE = 44 ms; flip angle 12°; matrix 256 × 256; FOV = 256 mm, voxel size = 1 mm× 1 mm× 1 mm. Data from one volunteer were excluded from analyses due to acquisition limitations resulting in incomplete brain coverage. The final dataset included 8 subjects.

MDMA

3,4-methylenedioxymethamphetamine (MDMA) combines the subjective effects of a stimulant and a psychedelic, inducing powerful euphoria and prosociality, but also mild visual hallucinations(*173–175*). It inhibits reuptake of noradrenaline, dopamine, and serotonin by acting on their respective transporters, and it also stimulates their release – with preferential effects on 5-HT mediating at least in part its induction of positive mood(*173–175*).

MDMA dataset: Recruitment

The MDMA dataset has been published before(*51*, *176*), and we refer the reader to the original publications for details. The study was approved by the National Research Ethics Service West London Research Ethics Committee, Joint Compliance and Research Office of Imperial College London, Research Ethics Committee of Imperial College London, Head of the Department of Medicine of Imperial College London, Imanova Centre for Imaging Science, and Faculty of Medicine of Imperial College London. The study was conducted in accordance with Good Clinical Practice guidelines. A Home Office Licence was obtained for the storage and handling of a Schedule 1 drug. Imperial College London sponsored the research.

The study included 25 healthy participants (mean age, 34±11 years; 7 females) with at least one previous experience with MDMA. None of the participants had used MDMA for at least 7 days or other drugs for at least 48 hours, which was confirmed by a urine screen. An alcohol breathalyzer test confirmed that none of the participants had recently consumed alcohol. Volunteers were screened for good physical and mental health, and magnetic resonance imaging

compatibility. All subjects were deemed physically and mentally healthy, and none had any history of drug or alcohol dependence or diagnosed psychiatric disorder.

MDMA dataset: Study protocol

The study design was within-subjects, and placebo-controlled. Participants were scanned twice, once after MDMA (100 mg encapsulated MDMA-HCl) and once after placebo (encapsulated ascorbic acid/vitamin-C), in a double-blind, randomized, counterbalanced order. The MDMA and placebo study days were separated by 7 days, and on each occasion, participants underwent two arterial spin labelling (ASL; not included in the present analysis) and two resting-state BOLD fMRI scans within a 90-minute scan session. Biochip Array Technology (Randox Laboratories Ltd., Co., Antrim, United Kingdom) was used to detect MDMA from plasma samples obtained shortly after each participant's MDMA scanning session (i.e., 2 hours after capsule ingestion).

The first resting-state BOLD scan took place 60 minutes after capsule ingestion and the second resting-state BOLD scan occurred 113 minutes after capsule ingestion. Participants relaxed with their eyes closed during the ASL and BOLD resting state scans. Peak subjective effects were reported ~100 minutes post administration of MDMA, consistent with the plasma t-max of MDMA. Therefore, here we focus on the second MDMA and placebo rs-fMRI scans.

MDMA dataset: MRI Data Acquisition

MR images were acquired on a 3T Siemens Tim Trio (Siemens Healthcare, Erlangen, Germany) using a 32-channel phased array head coil. Anatomical reference images were acquired using the ADNI-GO recommended MPRAGE parameters (1 mm isotropic voxels, TR = 2300 ms, TE = 2.98 ms, 160 sagittal slices, 256 x 256 in-plane resolution, flip angle = 9 degrees, bandwidth = 240 Hz/pixel, GRAPPA acceleration = 2). T2*-weighted echo-planar images (EPI) were acquired for the resting state BOLD functional scan using 3 mm isotropic voxels in a 192 mm in-plane FOV, TR = 2 s, echo time = 31 ms, 80 degree flip angle, 36 axial slices in each TR, bandwidth = 2298 Hz/pixel, and a GRAPPA acceleration of 2. Each rs-fMRI scan lasted for 6 minutes, corresponding to 180 functional volumes. One subject was excluded due to excessive artifacts in the functional MRI acquisition, leaving N=24 subjects for analysis.

Modafinil

Modafinil is a wakefulness promoting drug used for the treatment of sleep disorders such as narcolepsy (under the commercial name Provigil), as well as finding use as a cognitive enhancer for attention and memory(*5*, *53*, *177*), and to combat the cognitive symptoms of Attention Deficit/Hyperactivity Disorder (ADHD), and mood disorders, owing to its lower addiction risk in comparison with amphetamine-like psychostimulants(*5*, *177–180*). This drug has a broad neurotransmitter profile: it acts as a blocker of the dopamine and noradrenaline transporters, as well as modulating locus coeruleus noradrenergic firing, and acting on the wake-promoting hypothalamic neuropeptide orexin to activate the histamine system; it also influences both the glutamate and GABA systems(*178*, *181–185*).

Modafinil dataset: Recruitment

The modafinil dataset has been published before(*53*, *186*). As reported in the original publication, the study was approved by the ethics committee of University of Chieti (PROT

2008/09 COET on 14/10/2009) and conducted in accordance with the Helsinki Declaration. The study design was explained in detail and written informed consent was obtained from all participants involved in our study. Recruitment was performed throughout February 2011, drug/placebo administration and fMRI acquisitions started on March 2011, went on until January 2012, and the study was completed with the last fMRI session in January 2012. After securing financial coverage for costs related to the analysis of the study, the trial was registered on 10/09/2012 (NCT01684306http://clinicaltrials.gov/ct2/show/NCT01684306). After obtaining registration, the double-blind study was opened and analyzed.

This dataset was obtained from the OpenfMRI database. Its accession number is ds000133. A total of twenty six young male right-handed (as assessed by the Edinburgh Handedness inventory) adults (age range: 25–35 years) with comparable levels of education (13 years) were enrolled. All subjects had no past or current signs of psychiatric, neurological or medical (hypertension, cardiac disorders, epilepsy) conditions as determined by the Millon test and by clinical examination. Subjects showing visual or motor impairments were excluded as well as individuals taking psychoactive drugs or having a history of alcohol abuse. All volunteers were instructed to maintain their usual amount of nicotine and caffeine intake and avoid alcohol consumption in the 12h before the initiation of the study.

Modafinil dataset: Study protocol

Study subjects received, in a double blind fashion, either a single dose of modafinil (100 mg) (modafinil group; N=13) or a placebo (placebo group; N=13) pill identical to the drug. Randomization of study subjects was obtained by means of a random number generator. Here, we only considered data from the modafinil group. The day after drug/placebo assumption, subjects were asked about perceived side effects and, in particular, sleep disturbances. All but one reported no modafinil-induced side-effects or alterations in the sleep-wake cycle. Rs-fMRI BOLD data were separated in three runs lasting four minutes each followed by high resolution T1 anatomical images. Two scanning sessions took place: one before ingesting the drug/placebo, and one 3 hours later, to account for pharmacokinetics. Subjects were asked to relax while fixating the central point in the middle of a grey-background screen that was projected on an LCD screen and viewed through a mirror placed above the subject's head. Subject head was positioned within an eight-channel coil and foam padding was employed to minimize involuntary head movements.

Modafinil dataset: MRI Data Acquisition

BOLD functional imaging was performed with a Philips Achieva 3T Scanner (Philips Medical Systems, Best, The Netherlands), using T2*-weighted echo planar imaging (EPI) free induction decay (FID) sequences and applying the following parameters: TE 35 ms, matrix size 64x64, FOV 256 mm, in-plane voxel size 464 mm, flip angle 75 degrees, slice thickness 4 mm and no gaps. 140 functional volumes consisting of 30 transaxial slices were acquired per run with a volume TR of 1,671 ms. High-resolution structural images were acquired at the end of the three rs-fMRI runs through a 3D MPRAGE sequence employing the following parameters: sagittal, matrix 256x256, FOV 256 mm,slice thickness 1 mm, no gaps, in-plane voxel size 1 mm x 1 mm,flip angle 12 degrees, TR = 9.7 ms and TE = 4 ms. Two subjects from the modafinil group were excluded from analysis due to acquisition limitations, leaving N=11 subjects for analysis.

Methylphenidate

Methylphenidate is used as a cognitive enhancer to treat the cognitive symptoms of ADHD and narcolepsy (under the name Ritalin)(*4*, *187*). Pharmacologically, it inhibits the reuptake of both dopamine and noradrenaline by blocking their transporters; although yet to be conclusively confirmed, there is also in vitro evidence suggesting an additional minor affinity of methylphenidate for the 5-HT1A receptor(*188–194*).

Methylphenidate dataset: Recruitment

The methylphenidate dataset used here has been published before(*6*, *36*). Unlike the other datasets included in this study, the methylphenidate data were not obtained from healthy controls, but rather from a cohort of patients suffering from traumatic brain injury (TBI). Volunteers with a history of moderate to severe traumatic brain injury (inclusion criteria: age 18–60 years and not recruited to more than three research studies within the calendar year) were referred from the Addenbrooke's Neurosciences Critical Care Unit Follow-Up Clinic, Addenbrooke's Traumatic Brain Injury Clinic and The Royal London Hospital Intensive Care Unit (see the original publication for details of patient injuries). The patients were sent a written invitation to take part in the study. All volunteers gave written informed consent before participating in the study.

Thirty-eight volunteers were recruited to the study; 17 (12 male, 5 female) into the TBI arm of the study and 21 (13 male, 8 female) into the healthy control (HC) arm of the study. Exclusion criteria included National Adult Reading Test (NART) <70, Mini Mental State Examination (MMSE) <23, left-handedness, history of drug/alcohol abuse, history of psychiatric or neurological disorders, contraindications for MRI scanning, medication that may affect cognitive performance or prescribed for depression, and any physical handicap that could prevent the completion of testing.

Our sample contains mostly patients with diffuse axonal injuries and small lesions. The patients were at least 6 months post TBI. Four sustained moderate TBI with a score of between 9 and 12 on the Glasgow Coma Scale (GCS) and 11 sustained severe TBI with a GCS score of 8 or below on presentation. The mean age of the patient group was 36 years (±13 years).

Methylphenidate dataset: Study protocol

The study consisted of two visits (separated by 2–4 weeks) for both groups of participants. The TBI volunteers were randomly allocated in a Latin square design to receive one of the two interventions on the first visit (a placebo tablet or 30 mg tablet of methylphenidate), and the alternate intervention on the second visit. The decision to use 30 mg of methylphenidate was based on comparable doses used in previous studies in healthy participants, as well as NICE guidelines for medication in adults (www.nice.org.uk) which stipulate that when methylphenidate is titrated for side effects and responsiveness in each individual subject, the dose should range from a minimum of 15 mg to a maximum dose of 100 mg. As the dose of methylphenidate was not calculated by the participant's body weight, an interventional dose at the lower end of the dose range was chosen. After a delay of 75 min to ensure that peak plasma levels of methylphenidate were reached, the volunteers completed an MRI scan which included both fMRI and structural image acquisition. The healthy controls attended their two fMRI assessments at the same time interval as the patients, but without any pharmacological intervention. Therefore, here we only considered the patient data (drug versus placebo).

Methylphenidate dataset: MRI Data Acquisition

MRI data were acquired on a Siemens Trio 3-Tesla MR system(Siemens AG, Munich, Germany). MRI scanning started with the acquisition of a localizer scan and was followed by a 3D high resolution MPRAGE image [TR 2,300 ms, Echo Time (TE) 2.98 ms, Flip Angle 9◦, FOV 256×256 mm2]. Diffusion Tensor Imaging (DTI) data (63non-collinear directions,b=1,000 s/mm2with one volume acquired without diffusion weighting (b=0), echo time 106ms, repetition time 1,700 ms, field of view 192×192 mm,2 mm3isotropic voxels) were also collected to investigate white matter integrity. Here, we did not analyse the DTI data.

Functional imaging data were acquired using an echo-planar imaging (EPI) sequence with parameters TR = 2,000 ms, TE = 30 ms, Flip Angle = 78◦, FOV 192×192 mm2, in-plane resolution 3.0×3.0 mm, 32 slices 3.0 mm thick with a gap of 0.75 mm between slices. Two patients were excluded from the analysis (one patient only attended one of the study sessions and the other had excessive movement artifacts in their fMRI scan), leaving N=15 patients for analysis.

Functional MRI preprocessing and denoising

Preprocessing

To maximise the uniformity between the different datasets included here, we elected to preprocess all datasets using the same pipeline, as opposed to relying on the different pipelines originally employed by the group that collected each dataset. For the same reason, we also applied the same denoising procedure across all datasets.

For each dataset and condition, we applied a standard preprocessing pipeline, which we have previously employed with pharmaco-MRI datasets comprising both anaesthetics and psychedelics(*13*, *39*, *83*, *134*), demonstrating its suitability for the present analysis. Preprocessing was performed using the CONN toolbox, version 17f (CONN; http://www.nitrc.org/projects/conn)(*195*) based on Statistical Parametric Mapping 12 (http://www.fil.ion.ucl.ac.uk/spm), implemented in MATLAB 2016a. The pipeline involved the following steps: removal of the first 10s, to achieve steady-state magnetization; motion correction; slice-timing correction; identification of outlier volumes for subsequent scrubbing by means of the quality assurance/artifact rejection software *art* (http://www.nitrc.org/projects/artifact_detect); normalisation to Montreal Neurological Institute (MNI-152) standard space (2 mm isotropic resampling resolution), using the segmented grey matter image from each volunteer's T1-weighted anatomical image, together with an *a priori* grey matter template.

Denoising

Denoising was also performed using the CONN toolbox, using the same approach as in our previous publications with pharmaco-MRI datasets(*13*, *39*, *83*, *134*), which has also been adopted with similar pharmaco-MRI datasets in publications by independent groups(*196*).

Pharmacological agents can induce alterations in physiological parameters (heart rate, breathing rate, motion) or neurovascular coupling(*197*). The anatomical CompCor (aCompCor) method removes physiological fluctuations by extracting principal components from regions unlikely to be modulated by neural activity; these components are then included as nuisance regressors(*198*). Following this approach, five principal components were extracted from white matter and cerebrospinal fluid signals (using individual tissue masks obtained from the T1-weighted structural MRI images)(*196*); and regressed out from the functional data together with

six subject-specific realignment parameters (three translations and three rotations) as well as their first-order temporal derivatives; followed by scrubbing of outliers identified by ART, using Ordinary Least Squares regression(*195*). Finally, the denoised BOLD signal timeseries were linearly detrended and band-pass filtered to eliminate both low-frequency drift effects and high-frequency noise, thus retaining frequencies between 0.008 and 0.09 Hz.

The step of global signal regression (GSR) has received substantial attention in the literature as a denoising method(*199–201*). GSR mathematically mandates that approximately 50% of correlations between regions will be negative(*202*); however, the proportion of anticorrelations between brain regions has been shown to vary across states of consciousness, including anaesthesia and psychedelics(*13*, *83*). Furthermore, recent work has demonstrated that the global signal contains information about states of consciousness, across pharmacological and pathological perturbations(*203*). Therefore, in line with ours and others' previous studies, here we avoided GSR in favour of the aCompCor denoising procedure, which is among those recommended(*200*).

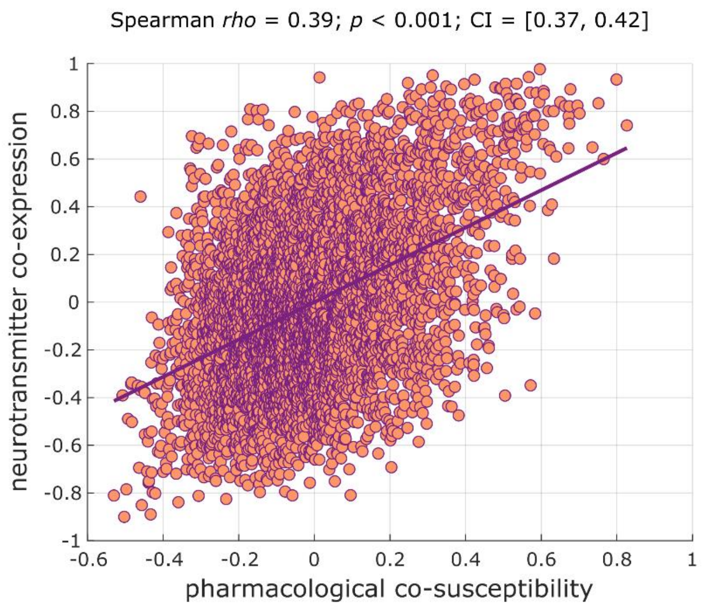

Fig. S1. Correlation between neurotransmitter co-expression and pharmacological co-susceptibility, after regressing out regional co-prevalence of non-grey matter tissue.

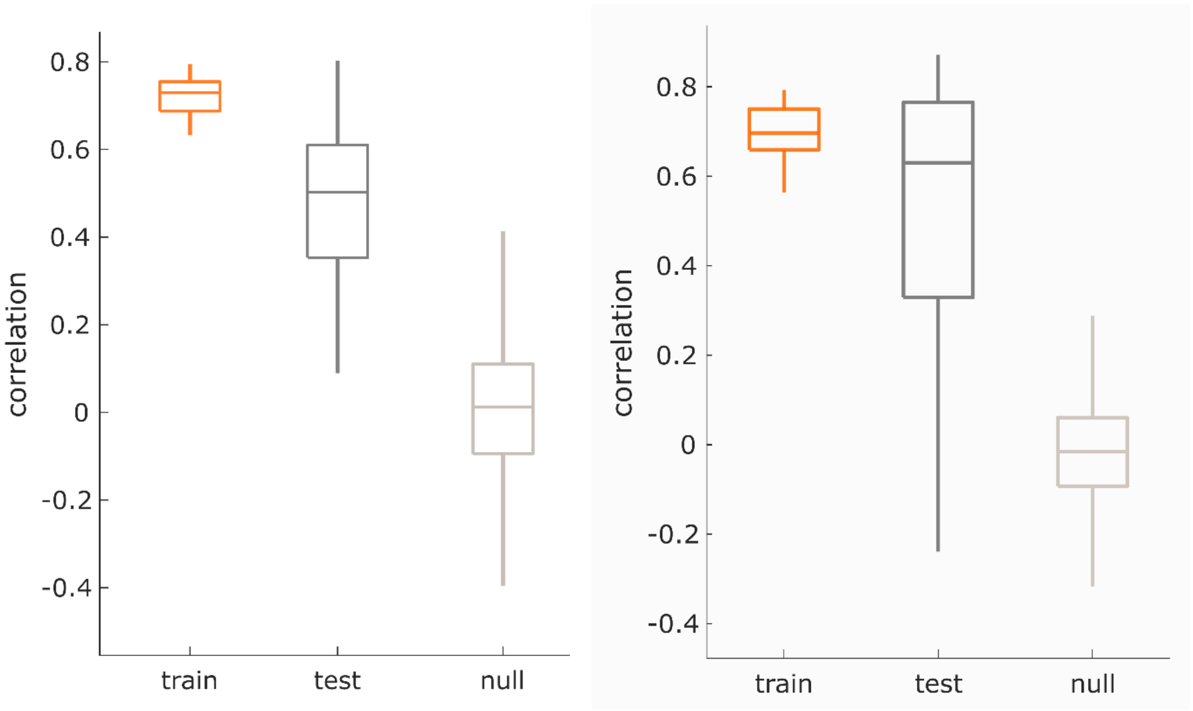

Fig. S2. Distance-dependent cross-validation. The correlation between drug scores and neurotransmitter scores was cross-validated by constructing the training set with 75% of brain regions closest in Euclidean distance to a randomly chosen source node (orange) and with the testing set as the remaining 25% of brain regions (dark grey; 100 repetitions). The out-of-sample mean was significant against a permuted null model (1,000 repetitions; null model shown in light grey; both $p < 0.001$). Left: PLS1, Right: PLS2.

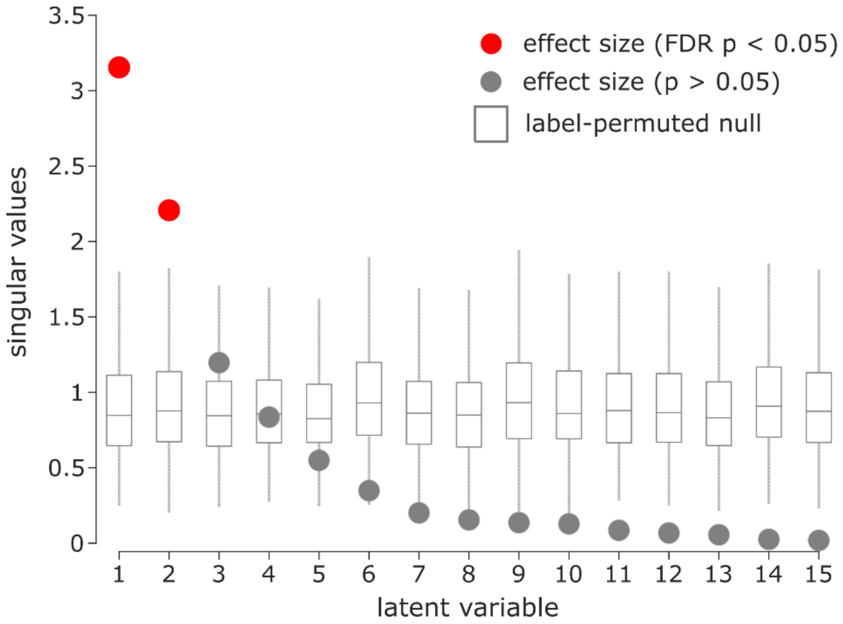

Fig. S3. Alternative null model for PLS analysis. A null distribution of singular values was derived by repeating the PLS analysis 1,000 times, with a null *Y* matrix obtained by randomly permuting subjects' drug and no-drug conditions. The p-values obtained against this null distribution represent the probability that the observed spatial correspondence between neurotransmitter density and drug-induced FC changes could occur by chance even in the absence of drug effects.

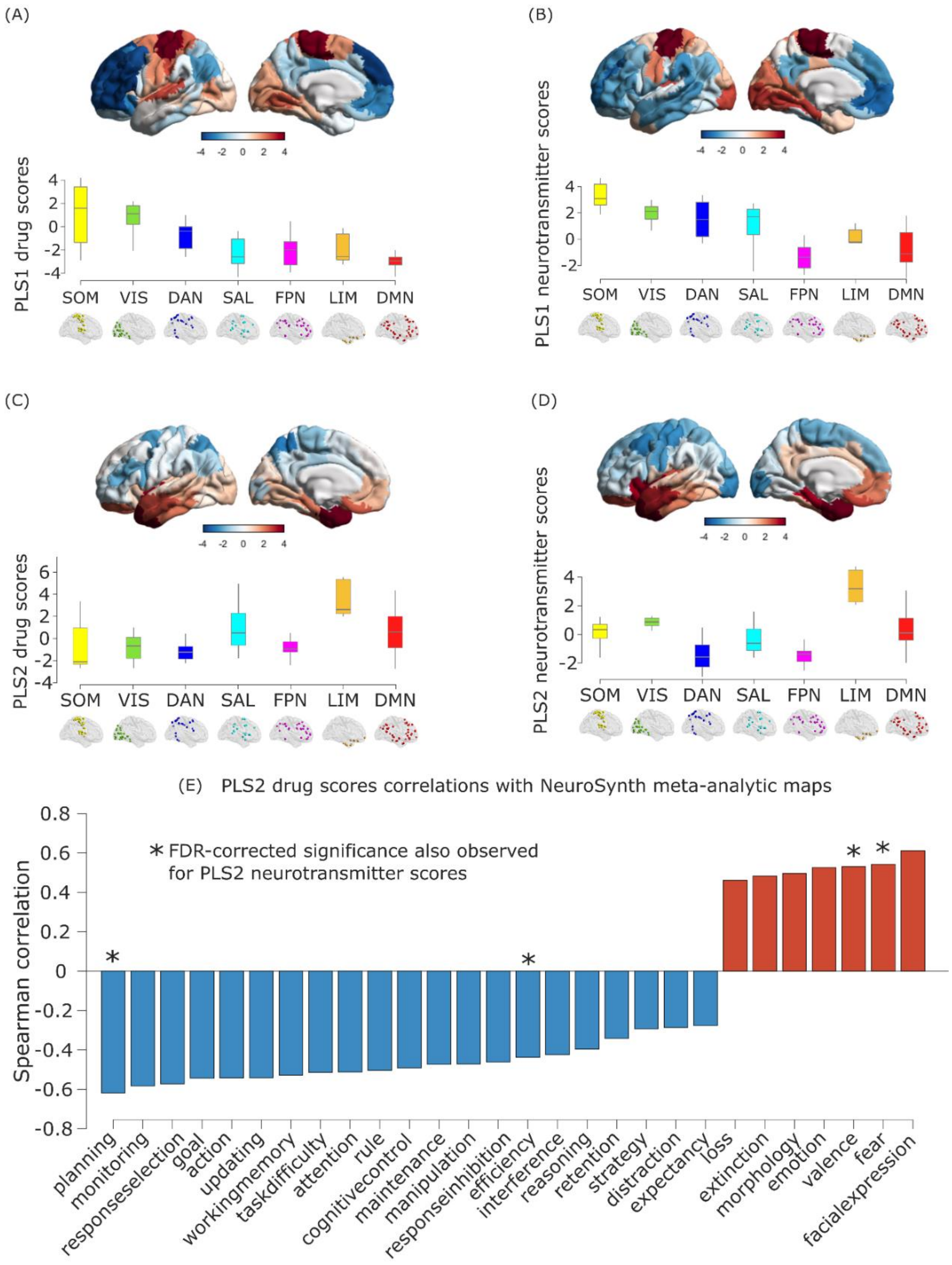

Fig. S4. Association between PLS1 and PLS2 scores and intrinsic resting-state networks. (A) PLS1 drug scores. (B) PLS1 neurotransmitter scores. (C) PLS2 drug scores. (D) PLS2 neurotransmitter scores. (E) Significant spatial correlations between PLS2 drug scores and 123 term-based meta-analytic maps from NeuroSynth. Significance is assessed against spatial autocorrelation-preserving null models, and corrected for multiple comparisons using the false discovery rate. * denotes correlations that are also significant (after FDR correction) for the PLS2 neurotransmitter scores.

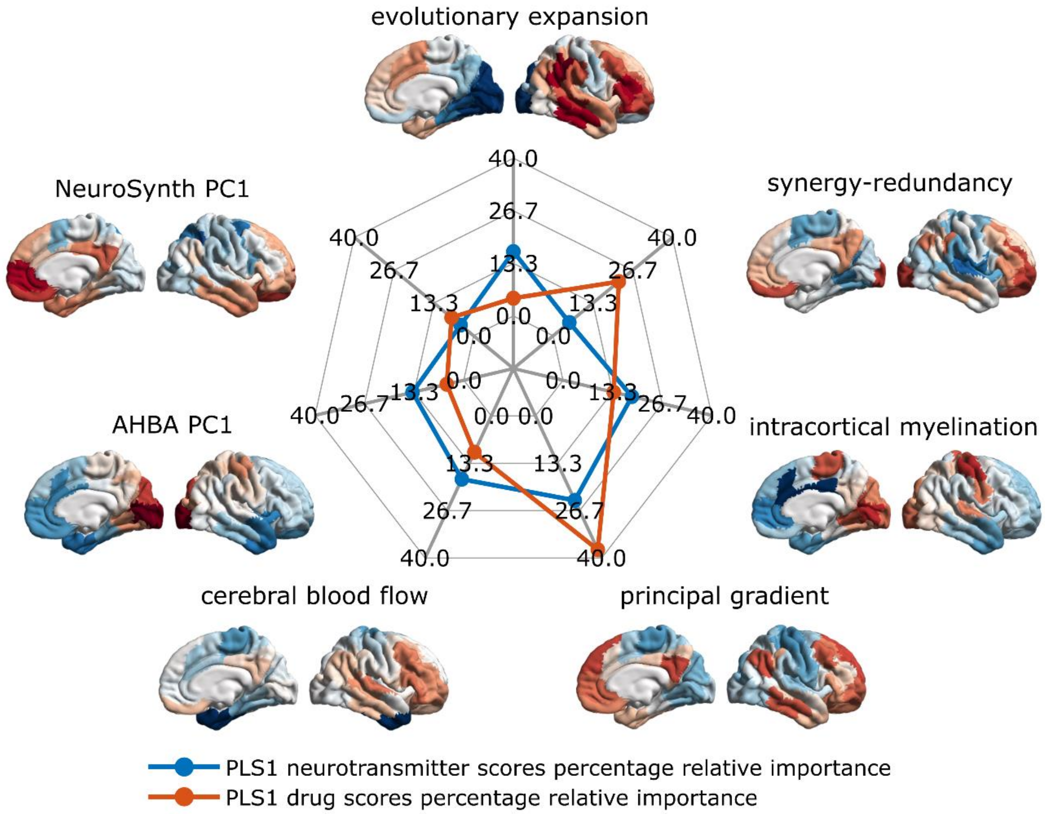

Fig. S5. Dominance analysis predicting PLS1 drug and neurotransmitter scores from all cortical hierarchies simultaneously. Spider-plots indicate the percentage of relative importance assigned to each predictor (cortical hierarchy), which together sum to 100%. Note that sign is not considered.

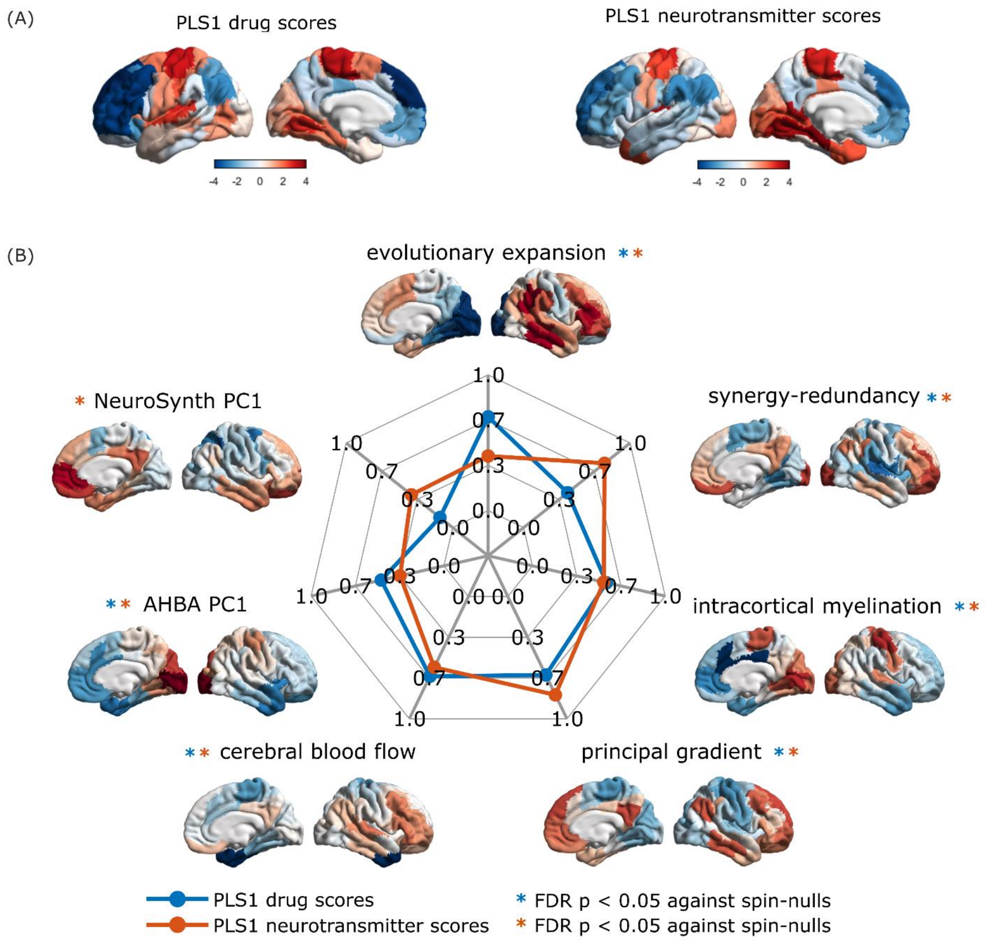

Fig. S6. Correspondence between the principal axis of drug-neurotransmitter scores and functional, anatomical and molecular hierarchies, after regressing out the regional probability of non-grey matter tissue. (A) Cortical distribution of drug and neurotransmitter scores for PLS1, after regressing out the regional probability of non-grey matter tissue. After regression, each pattern remains significantly correlated with the original scores (PLS1 drug scores: *rho* = 0.97, p_{spin} < 0.001; PLS1 neurotransmitter scores: *rho* = 0.90, p_{spin} < 0.001). (B) Radial plot represents the absolute value of the correlation between post-regression PLS1 drug and neurotransmiter scores, and each of seven cortical hierarchies obtained from different neuroimaging modalities (note that the myelin and AHBA PC1 maps are reversed with respect to the remaining hierarchies). *, FDR-corrected p_{spin} < 0.05.

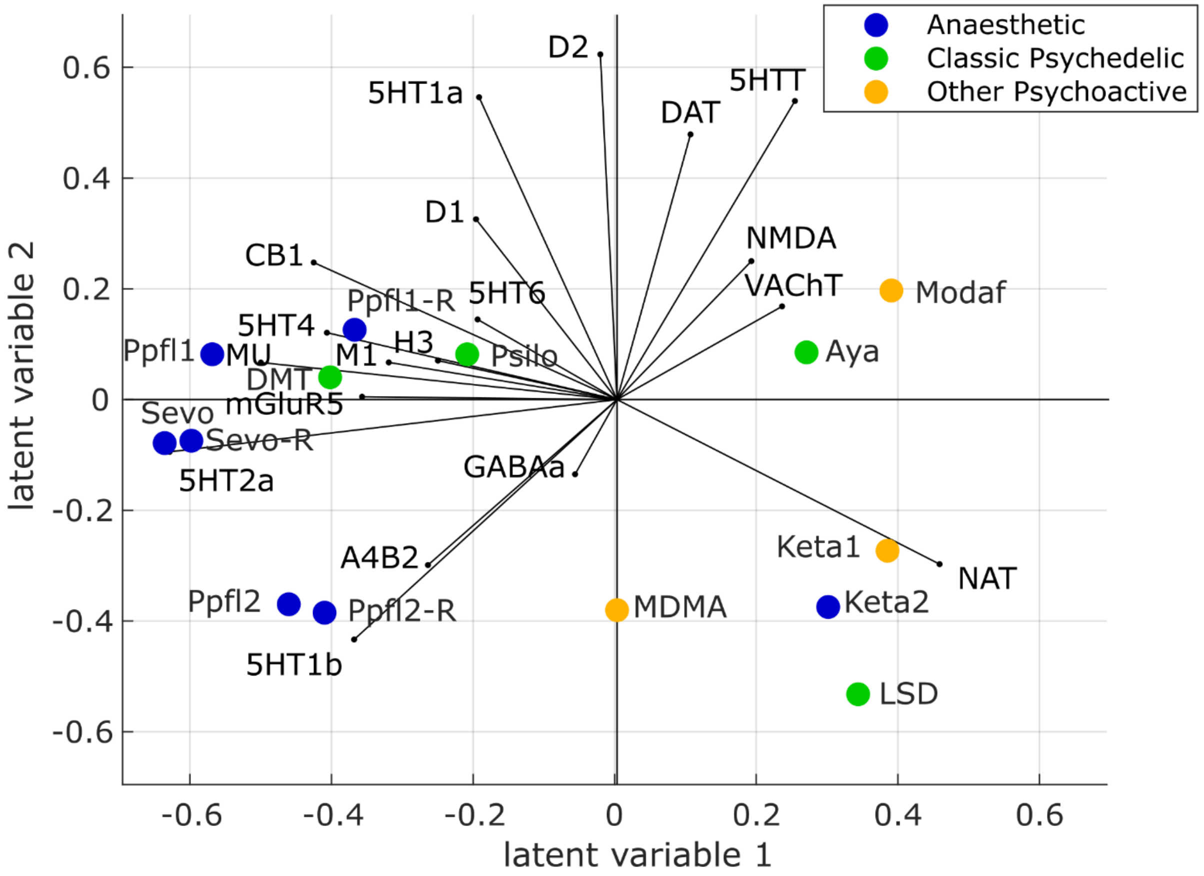

Fig. S7. Neurotransmitter landscape is qualitatively the same if methylphenidate data are not included. Each drug is represented as a point reflecting its projection onto the first two latent variables of the PLS analysis, color-coded based on its effects on subjective experience (anaesthetic, classic psychedelic, or other psychoactive). Each neurotransmitter receptor and transporter is represented as a vector in the same 2D space.

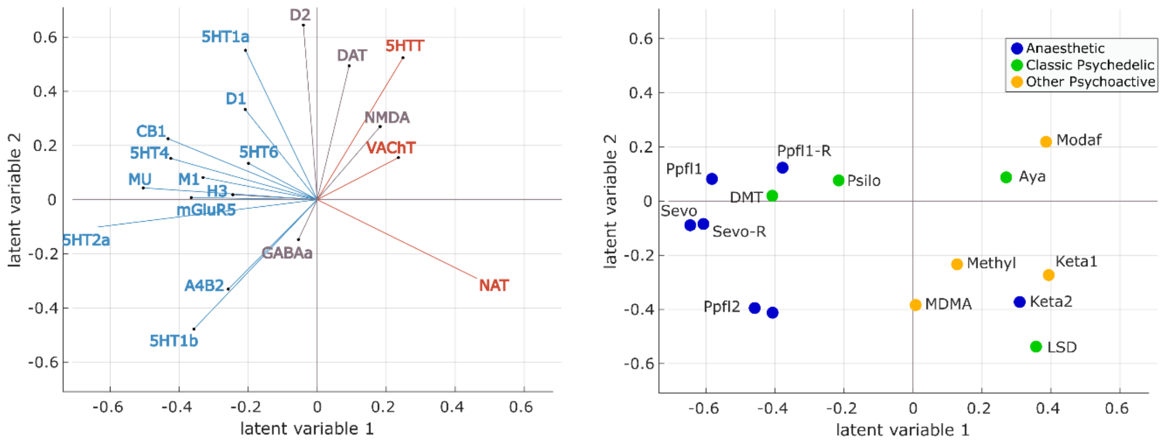

Fig. S8. Neurotransmitters and drugs plotted separately in the same space. Left: Each neurotransmitter receptor and transporter is represented as a vector in the 2D space of the first two PLS latent variables. Right: Each drug is represented as a point reflecting its projection onto the first two latent variables of the PLS analysis, color-coded based on its effects on subjective experience (anaesthetic, classic psychedelic, or other psychoactive).

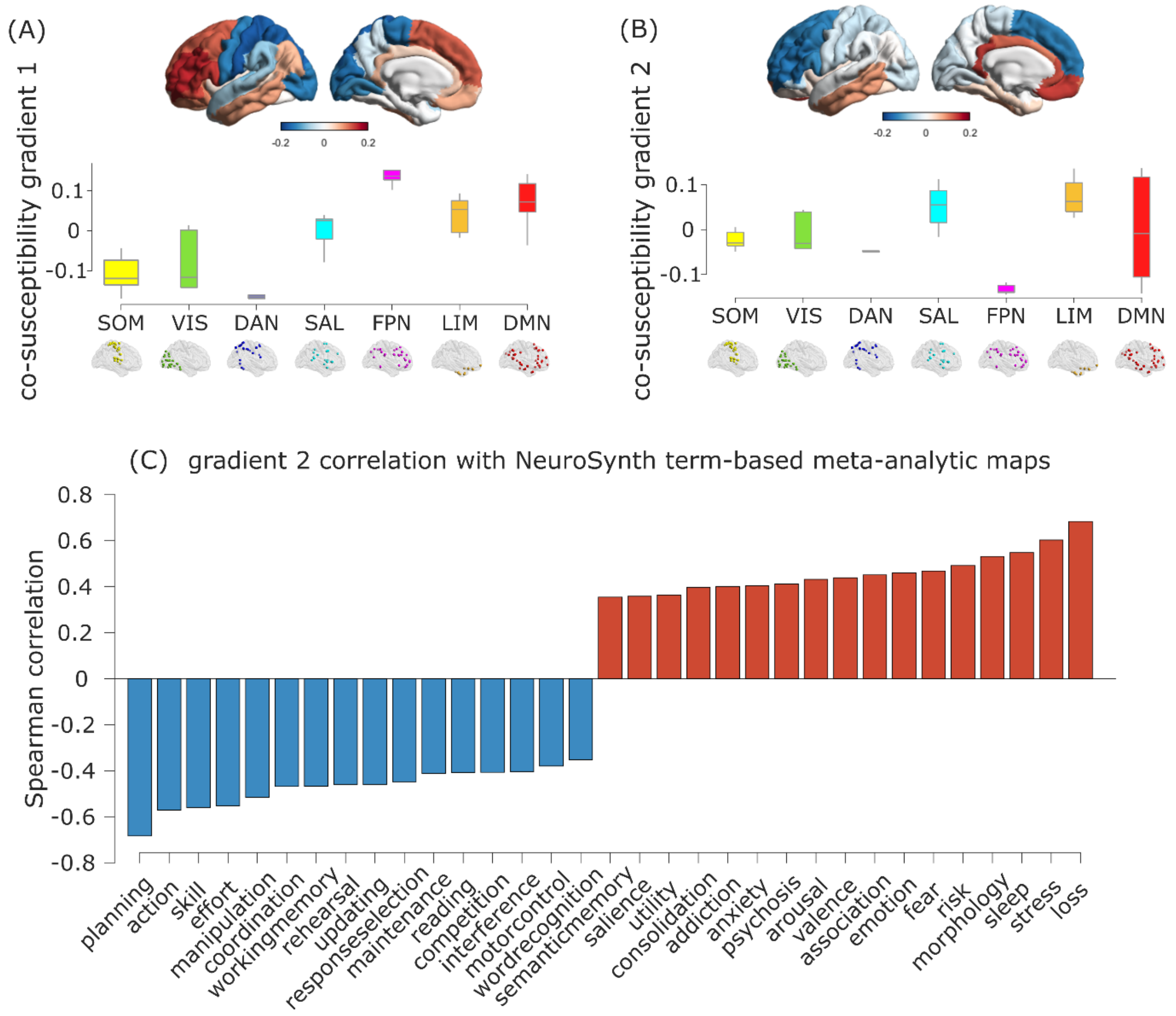

Fig. S9. Gradients of joint susceptibility to pharmacological and pathological perturbations and their association with intrinsic resting-state networks. (A) First joint gradient. There is a Spearman correlation of -0.89 (p = 0.012) between the signature of this gradient in terms of canonical resting-state networks, and the corresponding RSN signature of PLS1 drug scores from Fig. S4. For PLS1 neurotransmitter scores, the correlation coefficient is -0.71 (p = 0.088). (B) Second joint gradient. There is a Spearman correlation of 0.75 (p = 0.066) between the signature of this gradient in terms of canonical resting-state networks, and the corresponding RSN signature of PLS2 drug scores from Fig. S4. For PLS2 neurotransmitter scores, the correlation coefficient is -0.96 (p = 0.003). (C) Significant spatial correlations between the second joint gradient and 123 term-based meta-analytic maps from NeuroSynth. Significance is assessed against spatial autocorrelation-preserving null models, and corrected for multiple comparisons using the false discovery rate.

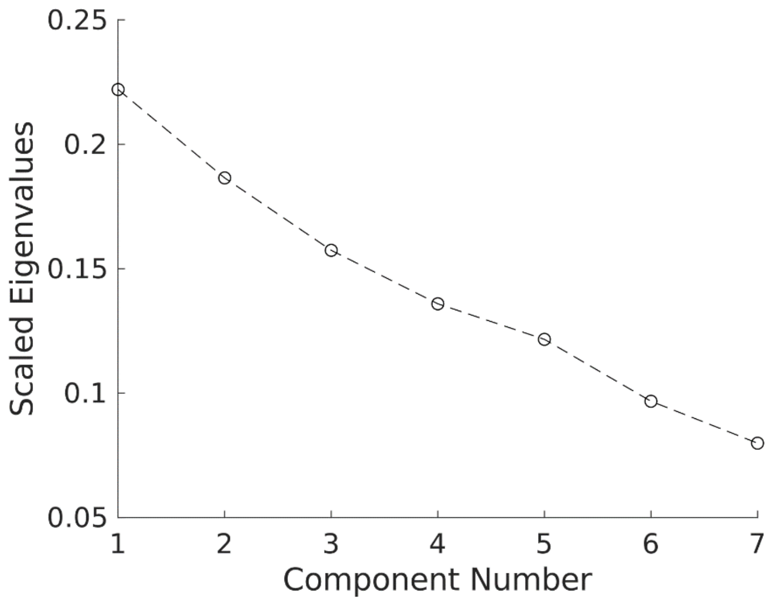

Fig. S10. Scree plot of the scaled eigenvalues from diffusion map embedding versus number of components, for the joint susceptibility gradients.

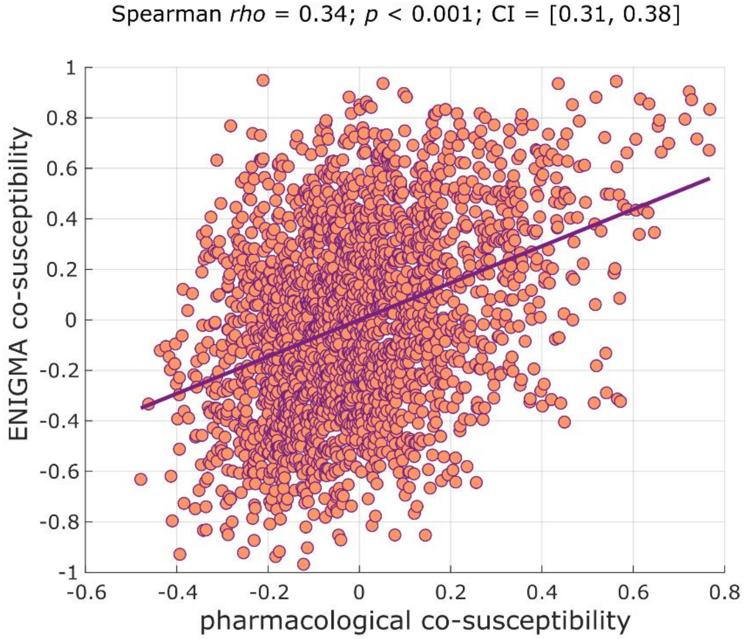

Fig. S11. Correlation between pharmacological co-susceptibility and co-susceptibility to disorder-related alterations of cortical thickness, after regressing out regional co-prevalence of non-grey matter tissue.

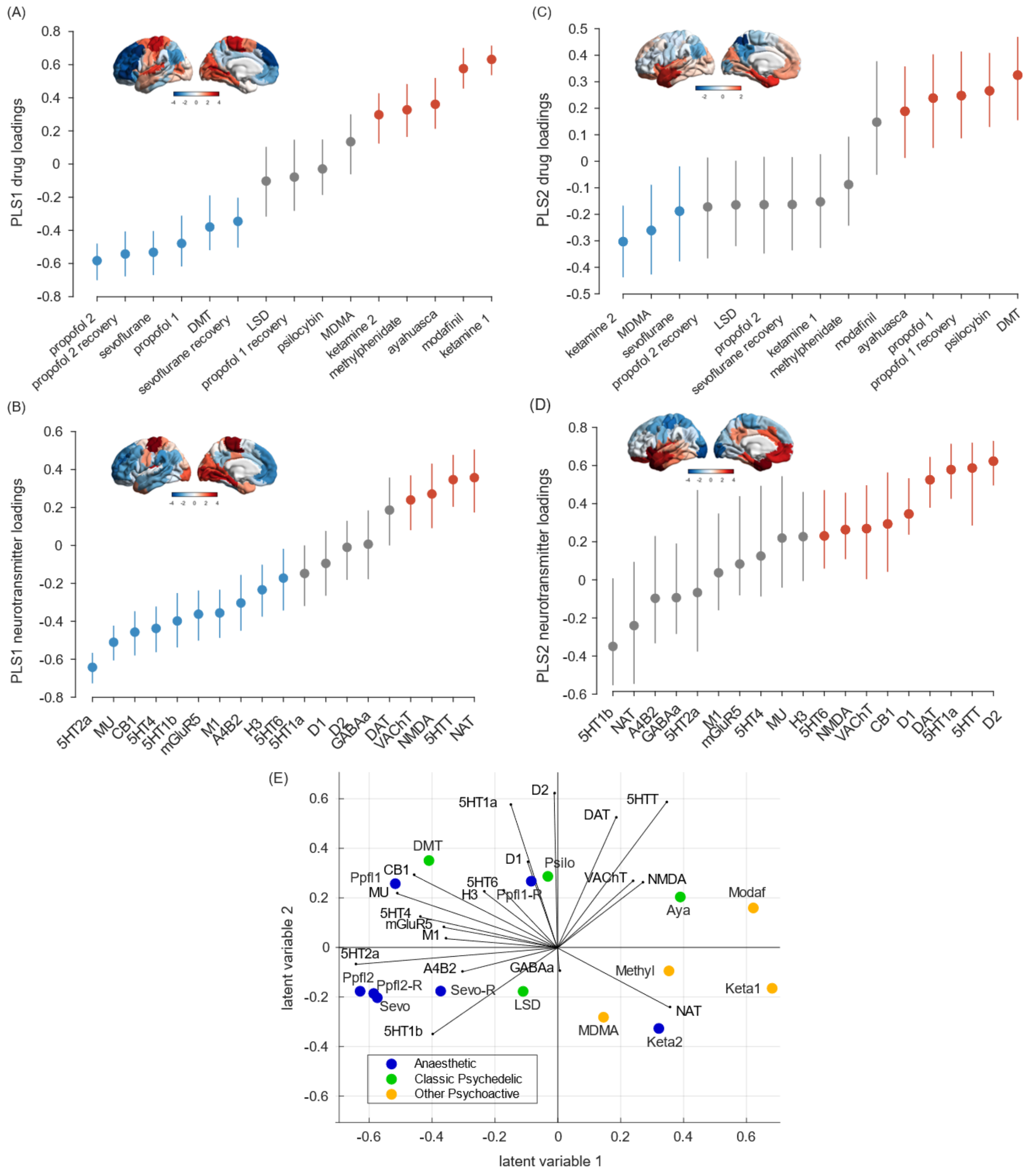

Fig. S12. Neurotransmitter-drug associations after additional motion regression. (A) PLS1 drug loadings and scores. (B) PLS1 neurotransmitter loadings and scores. (C) PLS2 drug loadings and scores. (D) PLS2 neurotransmitter loadings and scores. Brain maps show the z-scored cortical distributions of brain scores. (E) Biplot of neurotransmitters and pharmacological agents. Each drug is represented as a point reflecting its projection onto the first two latent variables of the PLS analysis, color-coded based on its effects on subjective experience (anaesthetic, psychedelic, or cognitive enhancer). Each neurotransmitter receptor and transporter is represented as a vector in the same 2D space.

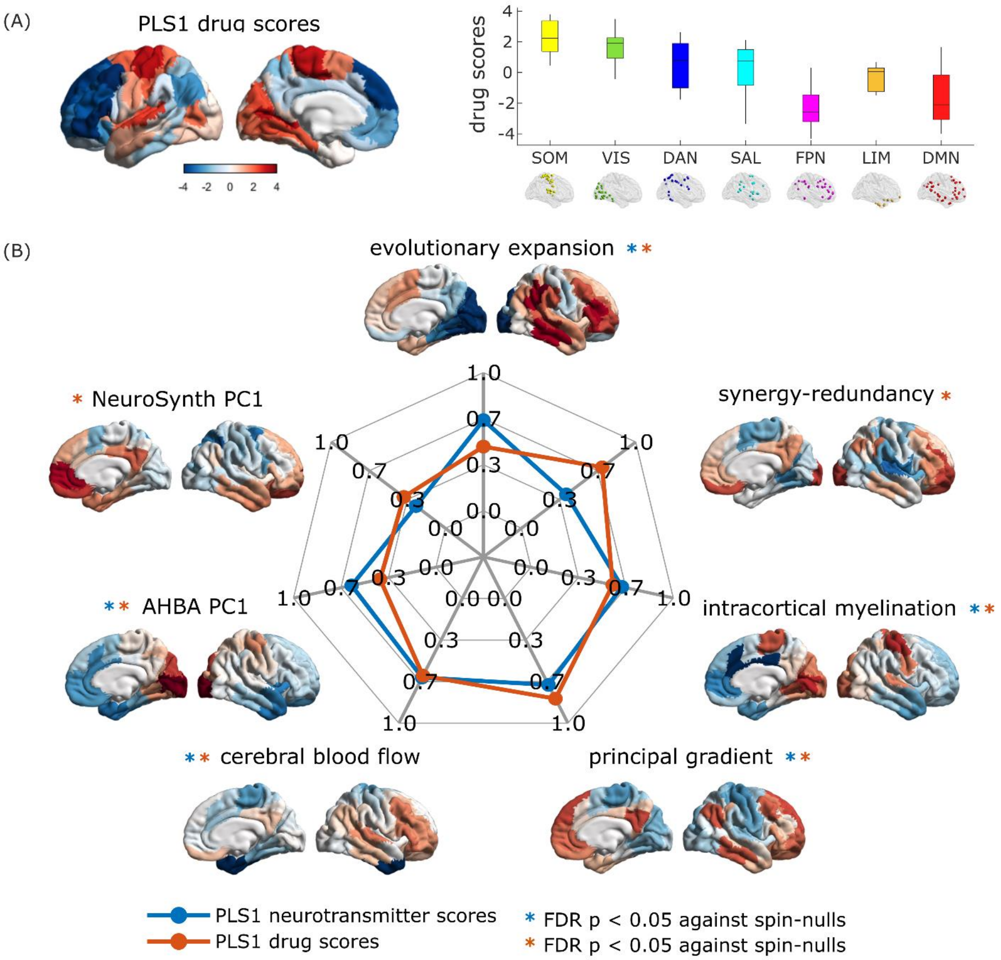

Fig. S13. Correspondence between the principal axis of drug-neurotransmitter scores and functional, anatomical and molecular hierarchies, after additional motion regression. (A) Cortical distribution of drug scores for PLS1 (z-scored), and breakdown into canonical resting-state networks. (B) Radial plot represents the absolute value of the correlation between PLS1 motion-regressed drug and neurotransmiter scores, and each of seven cortical hierarchies obtained from different neuroimaging modalities (note that the myelin and AHBA PC1 maps are reversed with respect to the remaining hierarchies). *, FDR-corrected $p_{spin} < 0.05$.

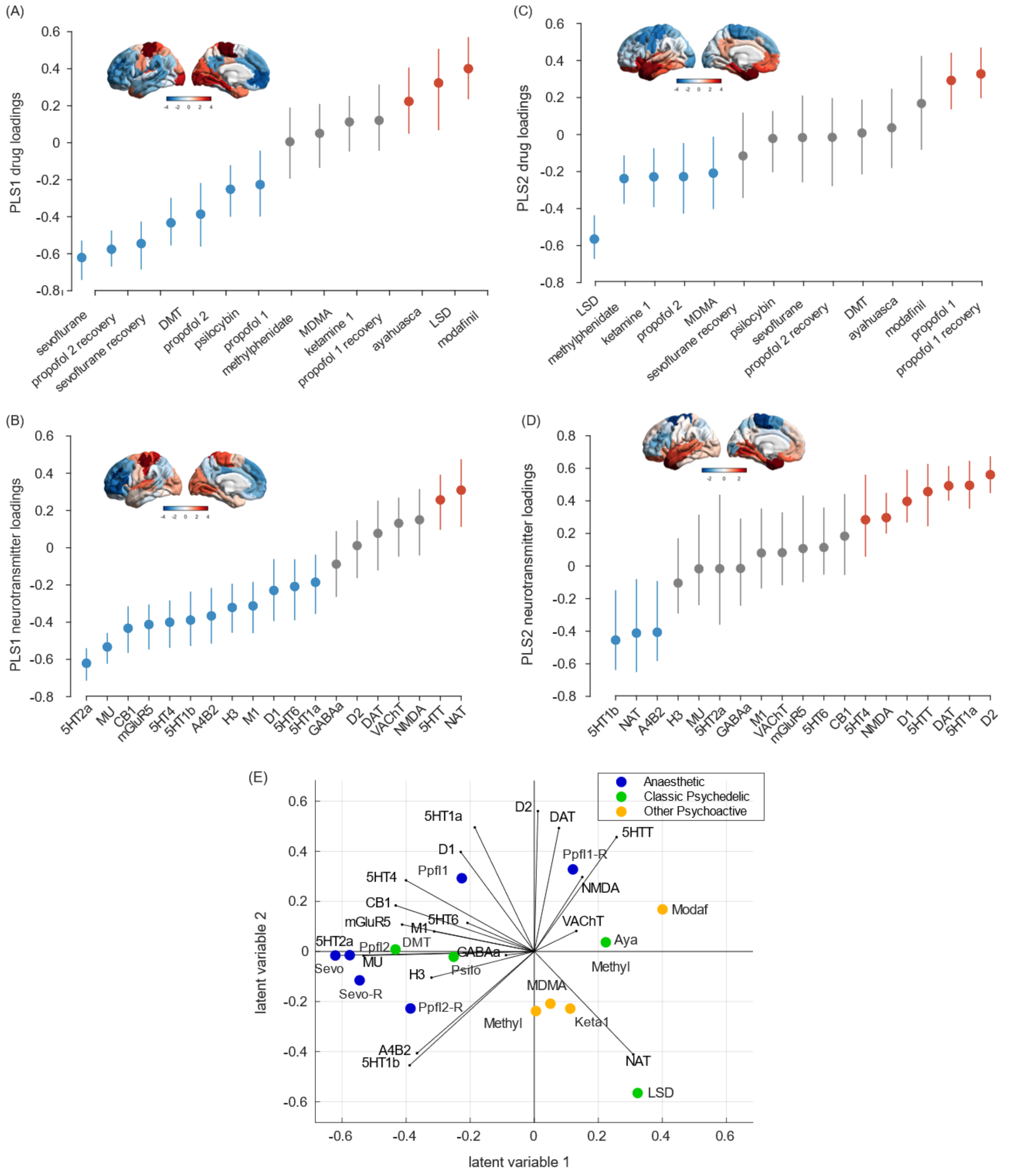

Fig. S14. Neurotransmitter-drug associations, with low-motion subjects only. (A) PLS1 drug loadings and scores. (B) PLS1 neurotransmitter loadings and scores. (C) PLS2 drug loadings and scores. (D) PLS2 neurotransmitter loadings and scores. Brain maps show the z-scored cortical distributions of brain scores. (E) Biplot of neurotransmitters and pharmacological agents. Each drug is represented as a point reflecting its projection onto the first two latent variables of the PLS analysis, color-coded based on its effects on subjective experience (anaesthetic, psychedelic, or cognitive enhancer). The anaesthetic ketamine dataset was not included for this analysis, as only one individual out of eight met the criterion in both scans. Each neurotransmitter receptor and transporter is represented as a vector in the same 2D space.

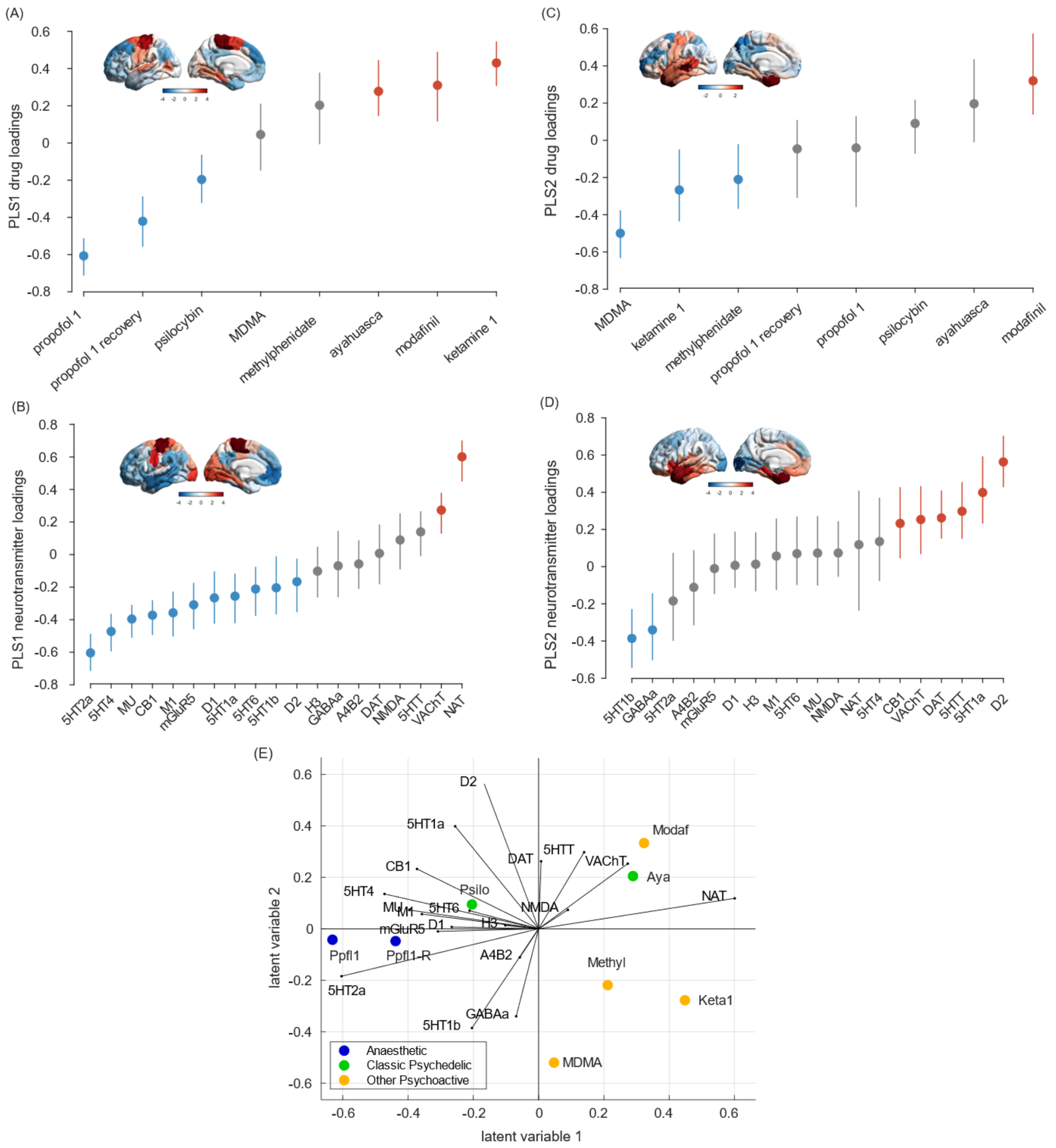

Fig. S15. Neurotransmitter-drug associations, for datasets without significant differences in motion. (A) PLS1 drug loadings and scores. (B) PLS1 neurotransmitter loadings and scores. (C) PLS2 drug loadings and scores. (D) PLS2 neurotransmitter loadings and scores. Brain maps show the z-scored cortical distributions of brain scores. (E) Biplot of neurotransmitters and pharmacological agents. Each drug is represented as a point reflecting its projection onto the first two latent variables of the PLS analysis, color-coded based on its effects on subjective experience (anaesthetic, psychedelic, or cognitive enhancer). Each neurotransmitter receptor and transporter is represented as a vector in the same 2D space.

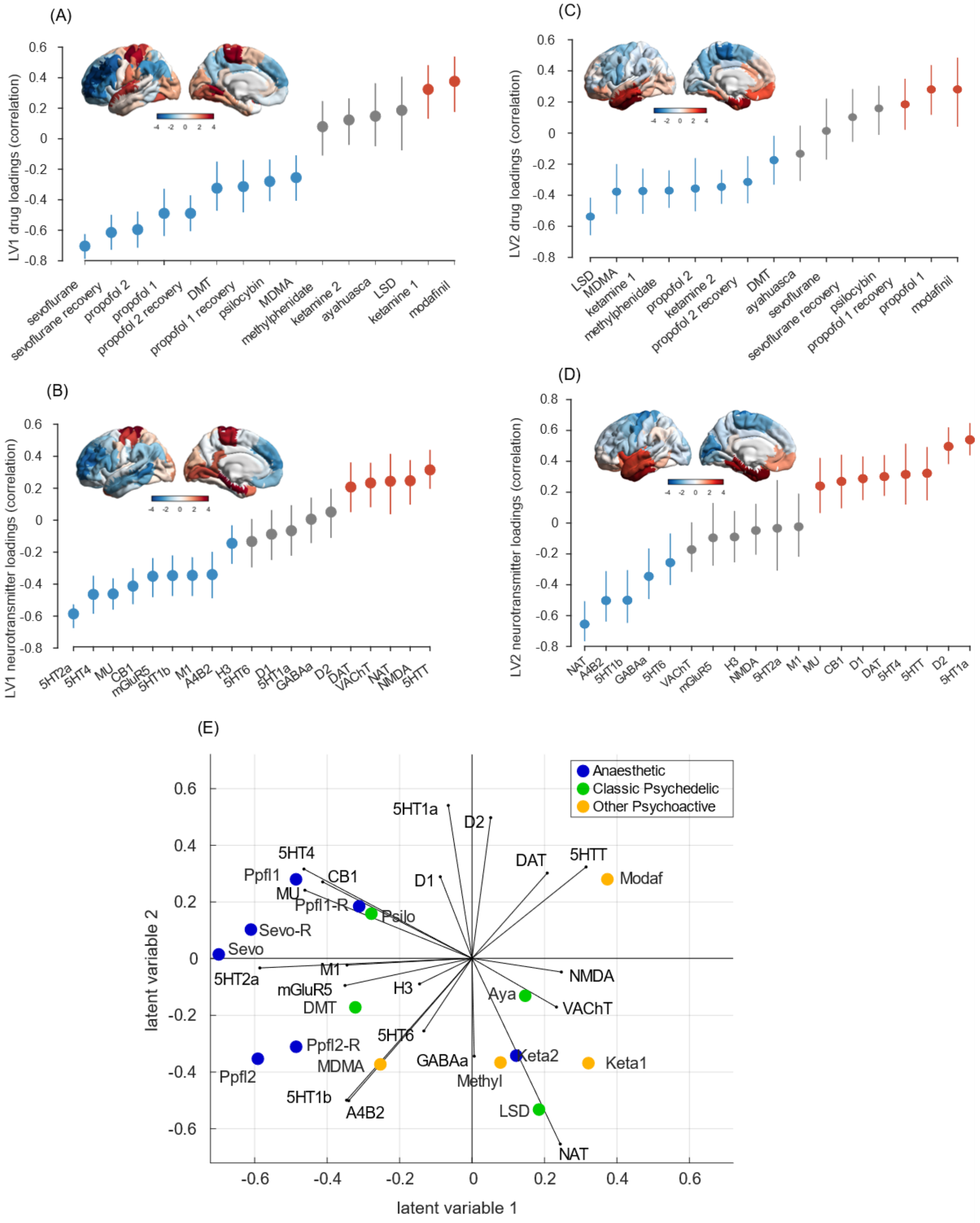

Fig. S16. PLS1 and PLS2 for Lausanne-114 cortical parcellation. (A) PLS1 drug loadings and scores. (B) PLS1 neurotransmitter loadings and scores. (C) PLS2 drug loadings and scores. (D) PLS2 neurotransmitter loadings and scores. Brain maps show the z-scored cortical distributions of brain scores. (E) Biplot shows neurotransmitters and pharmacological agents. Each drug is represented as a point reflecting its projection onto the first two latent variables of the PLS analysis, color-coded based on its effects on subjective experience (anaesthetic, classic psychedelic, or other psychoactive). Each neurotransmitter receptor and transporter is represented as a vector in the same 2D space.

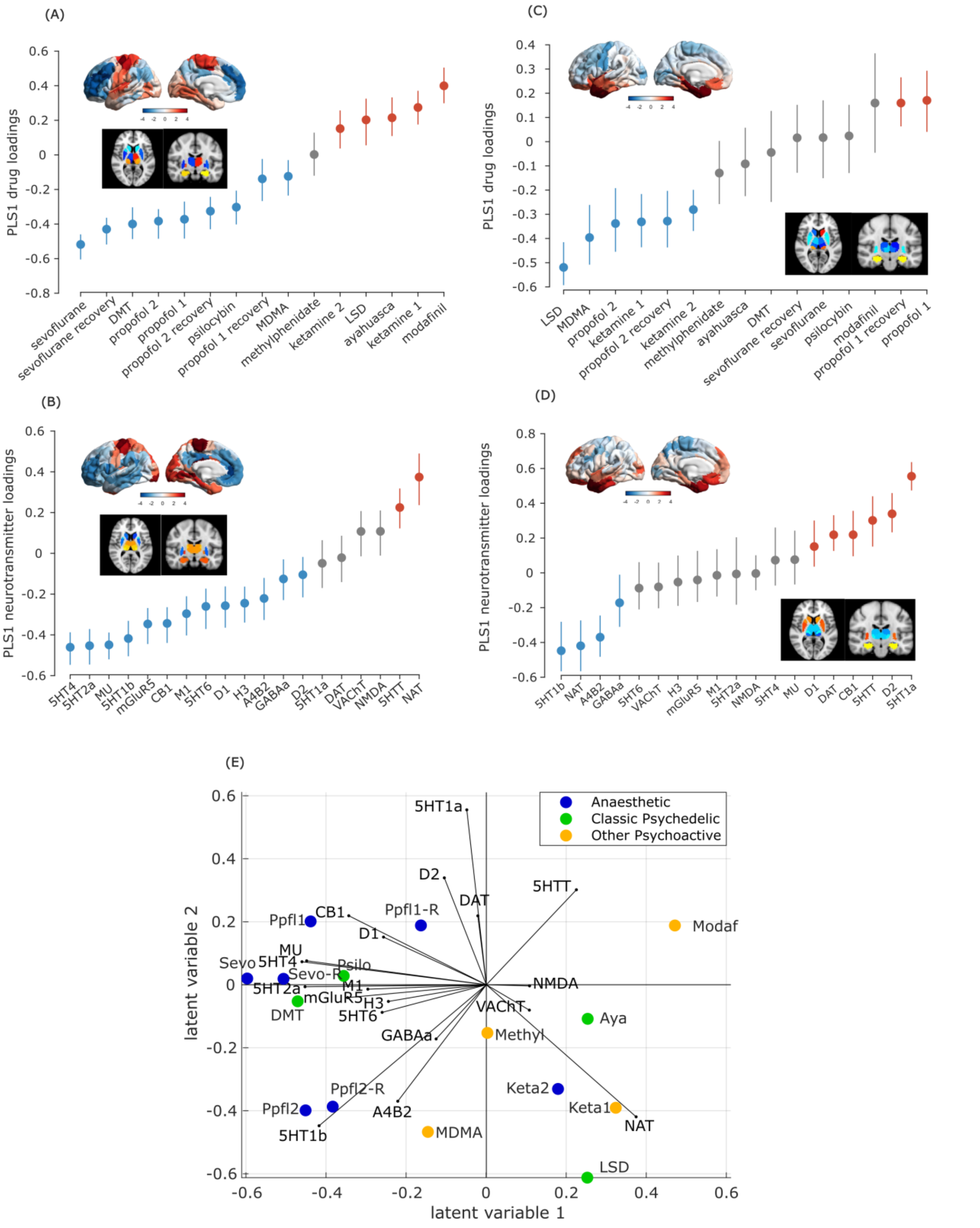

Fig. S17. Neurotransmitter-drug associations including both cortical and subcortical structures. (A) PLS1 drug loadings and scores, with subcortical PLS1 scores plotted on axial and coronal brain slices (32 ROIs from the Melbourne atlas). (B) PLS1 neurotransmitter loadings and scores, with subcortical PLS1 scores plotted on axial and coronal brain slices (32 ROIs from the Melbourne atlas). C) PLS2 drug loadings and scores, with subcortical PLS1

scores plotted on axial and coronal brain slices (32 ROIs from the Melbourne atlas). (D) PLS2 neurotransmitter loadings and scores, with subcortical PLS1 scores plotted on axial and coronal brain slices (32 ROIs from the Melbourne atlas). Brain maps show the z-scored cortical distributions of brain scores. (E) Biplot of neurotransmitters and pharmacological agents. Each drug is represented as a point reflecting its projection onto the first two latent variables of the PLS analysis, color-coded based on its effects on subjective experience (anaesthetic, psychedelic, or cognitive enhancer). Each neurotransmitter receptor and transporter is represented as a vector in the same 2D space.

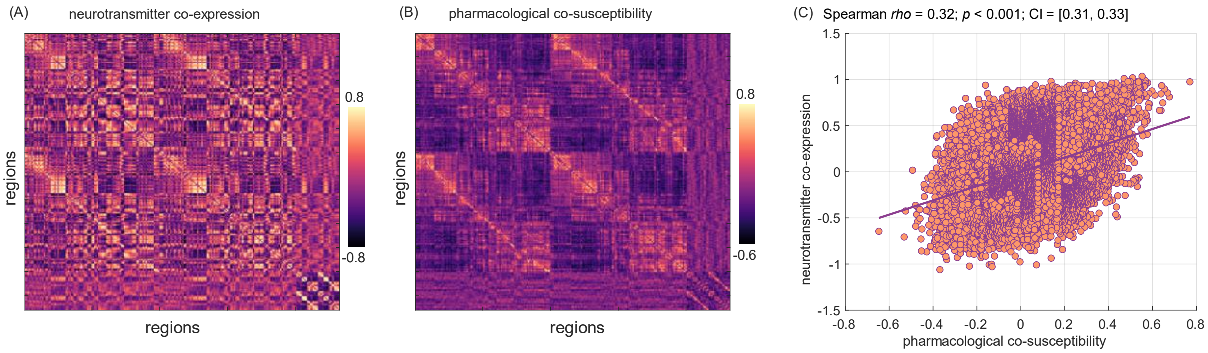

Fig. S18. Neurotransmitter co-expression (A) and pharmacological co-susceptibility (B) are significantly correlated (C) when both cortex and subcortex are included.

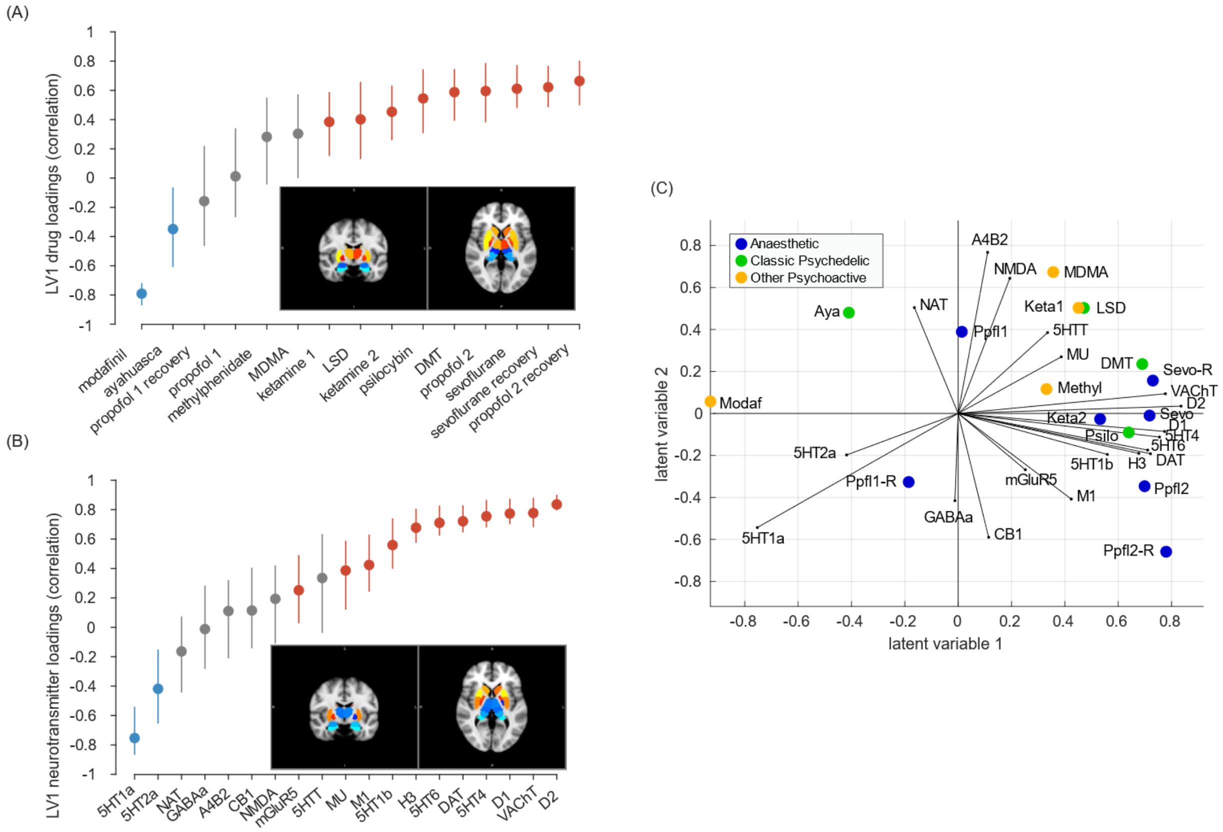

Fig. S19. Subcortical neurotransmitter-drug associations. (A) PLS1 drug loadings for PLS1, with subcortical PLS1 scores plotted on axial and coronal brain slices (32 ROIs from the Melbourne atlas(*107*)). (B) PLS1 neurotransmitter loadings, with subcortical PLS1 scores plotted on axial and coronal brain slices (32 ROIs from the Melbourne atlas(*107*)). (C) Biplot of neurotransmitters and pharmacological agents. Each drug is represented as a point reflecting its projection onto the first two latent variables of the PLS analysis, color-coded based on its effects on subjective experience (anaesthetic, psychedelic, or cognitive enhancer). Each neurotransmitter receptor and transporter is represented as a vector in the same 2D space.

	Healthy or patients	Within- or between-subjects design	Final N	Magnet strength	TR (s)	Duration (s)
Propofol1 (Cambridge)	Healthy	Within	15	3T	2.0	300
Propofol2 (Western)	Healthy	Within	16	3T	2.0	512
Sevoflurane	Healthy	Within	15	3T	1.838	700
Ketamine1 (sub-anaesthetic)	Healthy	Within	20	3T	2.0	600
Ketamine2 (anaesthetic)	Healthy	Within	8	3T	2.46	738
LSD	Healthy	Within	15	3T	2.0	440
Psilocybin	Healthy	Within	9	3T	3.0	300
DMT	Healthy	Within	13	3T	2.0	480
Ayahuasca	Healthy	Within	8	1.5T	1.7	255
MDMA	Healthy	Within	24	3T	2.0	360
Modafinil	Healthy	Within	11	3T	1.671	702
Methylphenidate	Patients	Within	15	3T	2.0	320

Table S1. Summary of pharmacological-MRI datasets.

	Mean (No Drug)	SD (No Drug)	Mean (Drug)	SD (Drug)	t-score	df	p-value
Propofol 1	0.26	0.13	0.34	0.14	-1.44	14	0.171
Propofol 1 - recovery	0.33	0.29	0.34	0.14	-0.08	14	0.937
Propofol 2	0.20	0.12	0.43	0.40	-2.85	15	0.012
Propofol 2 - recovery	0.18	0.06	0.43	0.40	-2.71	15	0.016
Sevoflurane	0.23	0.08	0.07	0.02	7.03	14	<0.001
Sevoflurane - recovery	0.19	0.08	0.07	0.02	5.75	14	<0.001
Ketamine 1	0.25	0.08	0.24	0.11	0.78	19	0.445
Ketamine 2	0.17	0.07	0.58	0.26	-4.18	7	0.004
LSD	0.08	0.03	0.13	0.04	-6.34	14	<0.001
Psilocybin	0.16	0.08	0.21	0.08	-1.26	8	0.243
DMT	0.14	0.03	0.19	0.05	-2.54	12	0.026
Ayahuasca	0.05	0.01	0.07	0.03	-1.67	7	0.138
MDMA	0.16	0.09	0.22	0.19	-2.05	23	0.052
Modafinil	0.20	0.08	0.18	0.05	0.78	10	0.454
Methylphenidate	0.23	0.24	0.22	0.20	0.10	14	0.921

Table S2. Statistics for mean framewise displacement for each contrast. Statistics are from permutation-based paired t-tests (10,000 permutations). SD, standard deviation; df, degrees of freedom.

Description of Auxiliary datasets file

The parcellated cortical maps of fMRI drug effects (groupwise deltas) and PET receptor/transporter density, are provided as an Auxiliary file in MATLAB format. Columns represent distinct maps, and rows are regions of the Schaefer-100 atlas.

Description of Supplementary References Excel file

Supplementary References are provided as an Excel file. References 113-203 pertain to the Supplementary Methods. References 204-247 correspond to the published studies underlying each neurotransmitter receptor and transporter PET map, and each of the cortical abnormality maps pertaining to neuropsychiatric, neurodevelopmental, and neurological diseases and disorders.
